# Regulation of germ cell development by ARI1 family ubiquitin ligases in *C. elegans*

**DOI:** 10.1038/s41598-018-35691-y

**Published:** 2018-12-10

**Authors:** Julian A. Poush, Nicolas A. Blouin, Kristin R. Di Bona, Vladimir Lažetić, David S. Fay

**Affiliations:** 1 Department of Molecular Biology, College of Agriculture and Natural Resources, University of Wyoming, Laramie, WY 82071 USA; 2 Wyoming INBRE Bioinformatics Core, Laramie, USA

## Abstract

RING-between-RING (RBR) E3 ubiquitin ligases are implicated in various developmental processes, and mutations in genes encoding RBR proteins HHARI/ARIH1 and Parkin are associated with human diseases. Here we show by phylogenetic analysis that the ARI1 family has undergone a dramatic expansion within the *Caenorhabditis* clade in recent history, a characteristic shared by some genes involved in germline development. We then examined the effects of deleting all ARI1 family members in the nematode *Caenorhabditis elegans*, which to our knowledge represents the first complete knockout of ARI1 function in a metazoan. Hermaphrodites that lacked or had strongly reduced ARI1 activity had low fecundity and were partially defective in initiation of oocyte differentiation. We provide evidence that the *C. elegans* ARI1s likely function downstream or in parallel to FBF-1 and FBF-2, two closely related RNA-binding proteins that are required for the switch from spermatogenesis to oogenesis during late larval development. Previous studies have shown that the E2 enzymes UBC-18/UBCH7 and UBC-3/CDC34 can functionally collaborate with ARI1 family members. Our data indicated that UBC-18, but not UBC-3, specifically cooperates with the ARI1s in germline development. These findings provide new insights into the functions of RING-between-RING proteins and Ariadne E3s during development.

## Introduction

The covalent modification of proteins with ubiquitin, a highly conserved 76-amino-acid polypeptide, is essential for the proper execution of a wide range of cellular and developmental functions^1,2^. Attachment of a single ubiquitin molecule to target substrates (mono-ubiquitination) can direct changes in protein trafficking, localization, stability, and activity^3^. Alternatively, ubiquitin chains (poly-ubiquitination) can be built by covalently linking the C-terminus of one ubiquitin to any of seven lysines of another ubiquitin molecule. Ubiquitin chains linked through Lys-48 typically marks substrates for degradation by the 26S proteasome^1,4^. Both mono- and poly-ubiquitination are reversible through the actions of substrate-specific proteases, providing additional levels of control and flexibility^5,6^.

Ubiquitin modification is accomplished by several enzymatic activities acting in a serial manner^7,8^. First, a ubiquitin-activating enzyme (E1) transfers a single molecule of ubiquitin to an active-site cysteine residue within a ubiquitin-conjugating enzyme (E2), creating a thioester bond. Next, the modified E2, in association with a ubiquitin ligase (E3), transfers the ubiquitin to a lysine residue on the target protein, generating an isopeptide bond. Several distinct biochemical mechanisms have been described for the modification of substrates by E2–E3 complexes, with E3s conferring most or all of the substrate specificity. In addition, the generation of poly-ubiquitin chains can in some cases require the actions of a ubiquitin assembly factor (E4)^9^.

About 165 monomeric-type E3 ligases are encoded by the *C. elegans* genome, which include members of the HECT, RING finger, U-box, and RING-between-RING (RBR) families^10^. In addition, *C. elegans* has the potential to express a large number of distinct multi-subunit E3s. These include several versions of the anaphase-promoting complex (APC) as well as cullin-based E3s such as Skp1–Cullin–F-box–RBX1/2 (SCF) complexes. Notably, the presence of ~25 Skp1-like proteins and >300 F-box family members raises the possibility that *C. elegans* may deploy a large number of SCF-type E3s^11^.

E3s are often categorized based on the mechanisms by which they transfer ubiquitin to target substrates. In the case of HECT ligases, ubiquitin is first transferred from the E2 to an active-site cysteine in the E3 before being relocated to a target lysine on the substrate. In contrast, standard RING ligases mediate the transfer of ubiquitin directly from the E2 cysteine to the substrate lysine. RBR motif−containing proteins, which include members of the human homolog of *Drosophila* Ariadne (HHARI; also called ARIH1) subfamily, constitute an additional class of E3 ligases^12–15^. RBRs contain two RING motifs that are separated by an in between RING (IBR) domain. Biochemically, RBRs resemble the HECT ligases in that they form a thioester intermediate with ubiquitin prior to substrate modification at lysines^16,17^. Because RBRs contain RING domains, however, they are sometimes referred to as RING-HECT hybrids. It has also been shown that whereas HHARI catalyzes mono-ubiquitin modification^18,19^, other RBRs, such as HOIP^20–22^, generate linear ubiquitin chains. More recently, it has been shown that an HHARI–E2 complex can act in combination with SCF complex components to promote the poly-ubiquitination of substrates^19,23^. This type of close collaboration between two distinct E2–E3 complexes may be a unique feature of HHARI, although the extent to which this occurs is unknown.

*C. elegans* encodes 11 predicted RBR proteins including homologs of human HHARI, ARIH2, TRIAD1, Parkin, Dorfin, ARA54, and XAP3^24^. The three closest *C. elegans* relatives to human HHARI, ARI-1.1, ARI-1.2, and ARI-1.3 (ARI-1.1–3), share a high level of sequence identity to each other and are co-expressed in both somatic tissues and the germline^25–29^. In addition, a fourth HHARI-like protein, TAG-349, is also expressed in germline and somatic tissues^28,30,31^. UBC-18/UbcH7 is a conserved E2 partner of Ariadne E3s^17,25,32,33^. In addition, the *C. elegans* HHARI members (ARI-1.1–3) cooperate with UBC-18 to control an early step of pharyngeal morphogenesis^25,34^. More recently, in collaboration with others, we demonstrated that the regulation of pharyngeal development also involves the E2 enzyme UBC-3 along with several SCF complex members^23,35^. In this study, we have analyzed the consequences of inactivating multiple *C. elegans* HHARI family members. We find that ARI-1.1–3 and TAG-349 (the ARI1s) are collectively important for normal fertility and also cooperate with germline factors to promote the switch from spermatogenesis to oogenesis during *C. elegans* development.

## Results

### The Ariadne family in *Caenorhabditis*

**Figure 1:**
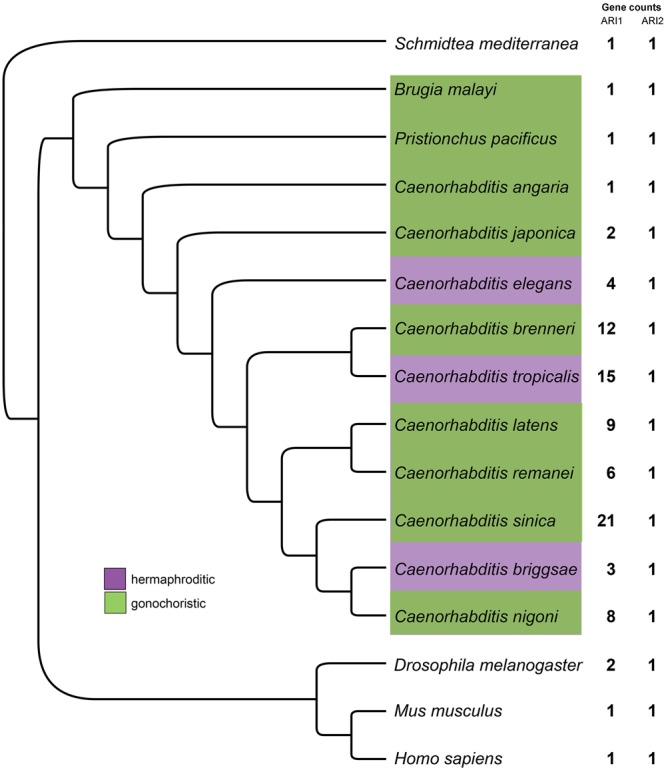
Species tree of ARI1 family members. Species tree showing relationships among the taxa whose Ariadne genes were included in our analysis. Numbers on the right of the figure indicate the number of *ari1* genes present in each of the genomes. Species positions were determined though whole-genome comparisons (see Materials and Methods).

To better understand the functions and evolution of Ariadne family members, we first carried out a phylogenetic analysis with a focus on the *Caenorhabditis* genus. Interestingly, whereas vertebrates and insects typically encode only one or two ARI1 family members, species within the *Caenorhabditis* genus have substantially increased copy numbers of this gene (Figs 1 and S1 and Supplementary File S1). This ranges from three *ARI1* members in *C. briggsae* to 21 in *C. sinica*. *ARI1* expansion was not observed in other assayed nematodes, such as the parasitic roundworm *B. malayi*, nor was duplication of *ARI2* family members generally detected (Figs 1 and S1). *ARI1* gene family size was not correlated with the mode of sexual reproduction and generally showed considerable variation within the assayed *Caenorhabditis* lineages (Figs 1 and S1). The Ariadne subfamily within the RBR ubiquitin ligases appears to be ancient, as it occurs in both unikont and bikont lineages^36,37^. Phylogenetic analysis of *Caenorhabditis ARI1* family members suggested that many of the observed duplications occurred following the split of *C. elegans* from other *Caenorhabditis* species and that many duplications of *ARI1* genes have occurred fairly recently in some species including *C. elegans* and, notably, *C. sinica* (Fig. S1). What is driving the duplication and potential sub-functionalization of the ARI1 genes is unknown; however, this is also seen with other RBR family members^36,37^.

**Figure 2:**
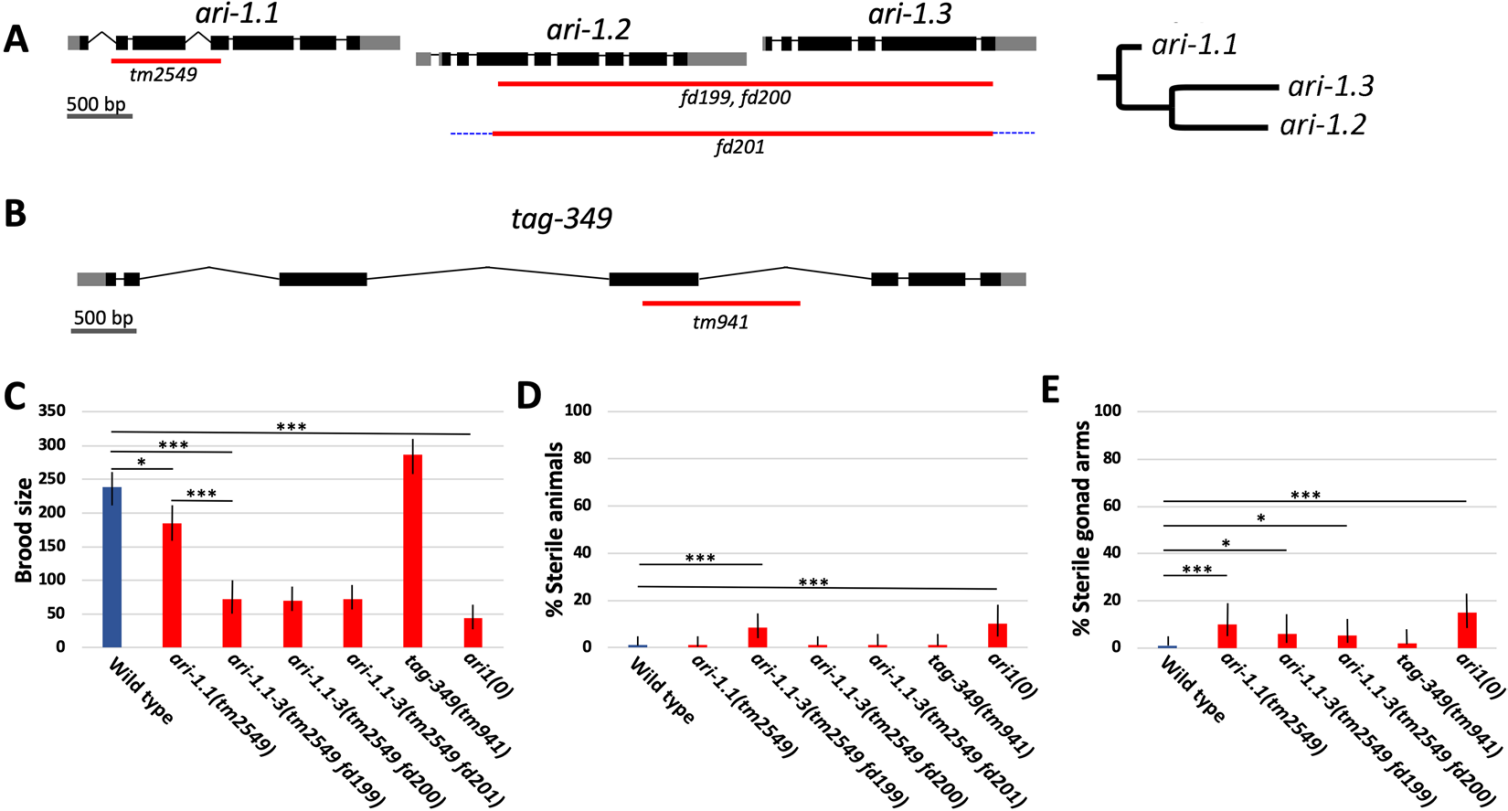
Compound deletions of *ari1* homologs reduce fecundity. (**A,B**) Gene diagrams of the (**A**) *ari-1.1–3* locus and (**B**) *tag-349* showing the extent of deletion mutations (red lines). Blue dashed line (*fd201*) indicates the presence of an incompletely characterized insertion. Dendrogram shows relationships between genes in the *ari-1.1–3* operon. (**C**–**E**) Quantification among wild type and *C. elegans ari1* single and compound mutants for (**C**) average brood size, (**D**) percentage of sterile animals, and (**E**) percentage of sterile gonad arms. Error bars (**C**–**E**) indicate 95% confidence intervals; *p < 0.05, ***p < 0.001.

In *C. elegans*, three of the ARI1 family members, *ari-1.1*/C27A12.8, *ari-1.2*/C27A12.7, and *ari-1.3*/C27A12.6 (ARI-1.1–3), are positioned in tandem within an ~7-kb region on LGI, where *ari1.1* is ancestral to *ari-1.2* and *ari-1.3* (Fig. 2A). These closely related homologs are predicted to make up an operon, with sequences upstream of *ari-1.1* driving the expression of all three genes. ARI-1.1–3 are 49–52% identical to HHARI at the amino acid level and are ~75% identical to each other, suggesting that their functions may be at least partially redundant^25^. Consistent with this, previous characterization of a null deletion allele of *ari-1.1*, *tm2549*, failed to uncover strong defects in growth, development, viability, or fertility^38^. *tag-349*/Y73F8A.34 encodes a fourth ARI1 member located on LG IV that is 46% identical to human HHARI and 58–60% identical to ARI-1.1–3. *tag-349* is a non-essential gene (see below) that is co-expressed with *ari-1.1–3* in the germline as well as in a number of somatic tissues^25,39^, consistent with the possibility that TAG-349 functionally collaborates with ARI-1.1–3.

### *C. elegans* ARI1 family members collectively promote normal fertility

To identify potential shared functions of C*. elegans* ARI1 family members, we used CRISPR/Cas9 methods to generate a triple deletion mutant of *ari-1.1–3*. The *ari-1.1(tm2549)* deletion removes 823 bp, including portions of intron 1 and exon 4, and is missing *ari-1.1* coding sequences corresponding to aa 23–198. Moreover, removal of the 3′ splice cite of intron 1 would be predicted to result in splicing between exon 1 and exon 5, leading to an in-frame deletion of aa 23–224. As this would result in the removal of the first RING finger (aa 127–180) and the N-terminal half of the IBR domain (aa 196–255), *tm2549* is expected to result in a full loss of ARI-1 function^15,38,40^. Using *ari-1.1(tm2549)* as a starting strain, we simultaneously targeted CRISPR/Cas9 cleavage sites bracketing *ari-1.2* and *ari-1.3*, leading to the isolation of three independent alleles (Fig. 2A). *fd199* is a 3791-bp deletion (LGI 6054173–6057963) along with a 25-bp insertion, whereas strain *fd200* is a 3988-bp deletion (LGI 6053993–6057980) with a 4-bp insertion. *fd201* is a 3850-bp deletion (LGI 6054118–6057967) along with a large insertion that was not fully characterized. Based on the extent of the deletions in *tm2549 fd199* and *tm2549 fd200* mutants, we would not expect these alleles to retain any residual ARI-1.1–3 activity and refer to these as *ari-1.1–3(0)*. Notably, we were able to propagate all three *ari-1.1–3* deletion strains as homozygotes, indicating that the ARI-1.1–3 homologs are collectively nonessential for viability under standard laboratory conditions.

Although the *ari-1.1–3* deletion strains were viable, we did observe that they were slower to deplete their bacterial food source than wild type, suggesting a reduction in growth rates or fecundity. Measurement of brood sizes indicated a ~70% reduction in all three *ari-1.1–3* deletion strains relative to wild type; average brood sizes ranged from ~70–73, although there was substantial variability between individuals (Table S1). In contrast, *ari-1.1(tm2549)* mutants showed only a modest reduction in brood size as compared with wild type (p = 0.03) Fig. 2C). *ari-1.1–3* deletion strains also showed a low but significant frequency of sterility, particularly when assayed per gonad arm (Fig. 2D,E). Nevertheless, the large majority of *ari-1.1–3* deletion animals produced some progeny. We note that gene-specific RNAi directed against single *ari-1.1–3* members is not feasible because of their high degree of similarity at the nucleotide level^25^.

*tag-349* encodes a fourth ARI1 homolog and is located on a separate chromosome (LGIV) from *ari-1.1–3*. *tm941* is a 775-bp deletion that removes a large portion of *tag-349* exon 4 and is predicted to result in the truncation of TAG-349 sequences following residue 231 of the 485-aa protein (Fig. 2B). Importantly, the translated product is predicted to lack the C-terminal 83 aa of the RBR domain as well as the Ariadne domain and thus should result in very strong or complete loss of function. *tag-349(tm941)* homozygotes are viable and fertile with brood sizes similar to that of wild type (p = 0.079) (Fig. 2C–E; Table S1).

We next generated a quadruple mutant strain (*tm2549 fd199; tm941*) (termed *ari1(0)*), which, although viable, had an average brood size of 44.3 (n = 18), the lowest among the assayed strains (Fig. 2C; Table S1). This compares to an average brood size of 71.1 for the *ari-1.1–3* deletions (p = 0.026; using pooled data from strains *tm2549 fd199*, *tm2549* fd200, and *tm2549 fd201*). We also observed a slightly higher percentage of sterile animals and a corresponding higher percentage of sterile gonad arms in *ari1(0)* animals relative to the *ari-1.1–3* deletion strains (Fig. 2D,E). For example, 15% of *ari1(0)* gonad arms were sterile versus 5.7% for *ari-1.1–3* strains (p = 0.015; using pooled data from strains *tm2549 fd199* and *tm2549 fd200*). Taken together, our findings indicate that the ARI1 homologs promote normal fecundity and perform functions that are at least partially overlapping. We also note that long-term passage of mutant strains including *tag-349*, *ari-1.1–3*, and *ari1(0)* led, in some cases, to substantially reduced fertility over time. Thus, we conducted experiments using strains that had been passaged for a minimal number of generations.

### *ari1* compound mutants exhibit germline defects

**Figure 3:**
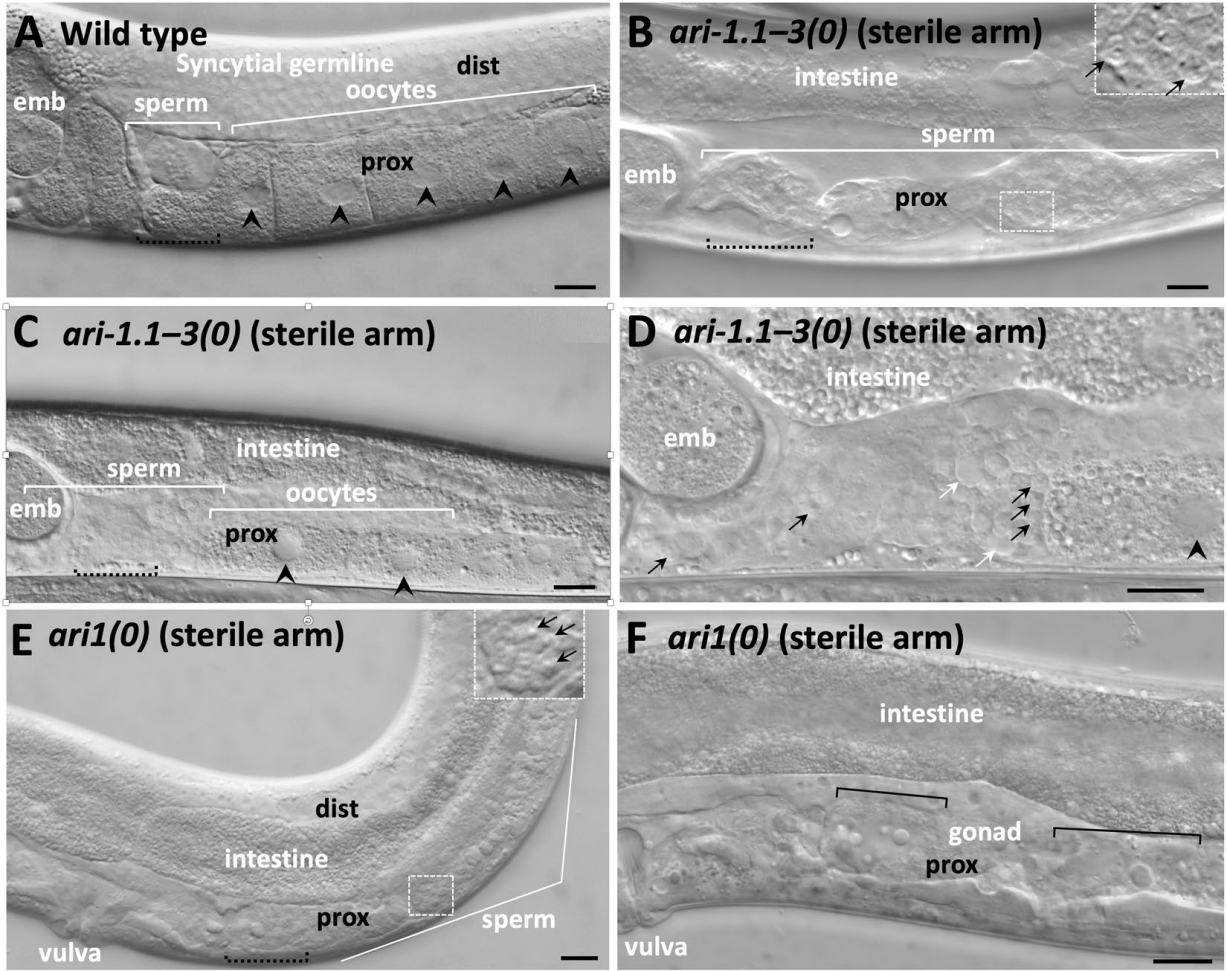
Images of gonads in wild type and in *ari1* mutant strains. (**A**–**F**) DIC images of gonad arms in (**A**) fertile wild-type, (**B**–**D**) sterile gonads arms of *ari-1.1–3(tm2549 fd199)*, and (**E,F**) sterile gonad arms of *ari-1.1–3(tm2549 fd199); tag-349(tm941)* adult hermaphrodites. Panels are oriented such that ventral is down and the vulva is to the left (visible in panels E and F only). Worms in (**E** and **F**) are sterile; (**B**–**D**) have a single sterile gonad arm (pictured). embryos (emb) present in the uterus in (**B**–**D**) are produced by the fertile gonad arm (not pictured). Proximal (prox) and distal (dist) regions of the gonad arm are labelled where visible; the distal portions in (**B**–**D**,**F)** is obscured by intestine. The approximate region of the spermatheca, where visible, is indicated by black dashed brackets. Black arrowheads (**A,C,D**) indicate oocyte nuclei; black arrows (**B,D,E**), sperm nuclei; white arrows (**D**), residual bodies; black brackets (**F**), regions that appear to have undergone germline deterioration or decomposition and may contain vacuoles are indicated by black brackets. Magnified insets (**B,E**) are indicated by dashed boxes. Scale bars (**A**–**F**) = 10 µm.

*C. elegans* hermaphrodites contain mirror-symmetric anterior and posterior gonadal arms, each capable of generating ~150 self progeny. During the fourth larval stage (L4), the hermaphrodite germline produces sperm exclusively, which becomes concentrated within a narrow proximal compartment of the somatic gonad termed the spermatheca^41,42^. Beginning at the adult stage, the hermaphrodite germline is reprogrammed to produce oocytes. Whereas sperm in wild-type hermaphrodites is usually confined to the spermatheca, the region containing sperm in sterile *ari-1.1–3* deletion animals was often expanded distally (also see below), and in some cases germlines failed to contain visible oocytes (Fig. 3A–D). In addition, we observed residual bodies, a byproduct of sperm differentiation^43^, in the sterile gonads of *ari-1.1–3* deletion adults (Fig. 3D). In contrast, residual bodies are normally not observed in adult-stage wild-type worms, which have completely switched over to oogenesis.

Inspection of sterile gonads in *ari1(0)* hermaphrodites revealed germline masculinization that was generally more severe than that observed in *ari-1.1–3* mutants (Fig. 3E), consistent with the reduced brood sizes of *ari1(0)* mutants as compared with *ari-1.1–3* deletion strains. Importantly, we did not observe obvious masculinization of somatic tissues in *ari-1.1–3(0)* or *ari1(0)* hermaphrodites, indicating that *C. elegans* ARI1 family members are likely to affect sex-specific differentiation within the germline only. We also observed variable germ cell deterioration within sterile *ari1(0)* gonad arms, including abnormal vacuoles, cellular debris, and aberrant cell morphologies (Fig. 3F). Our findings indicate that the *C. elegans* ARI1 proteins collectively promote oocyte development and may also have additional roles in germline health or maintenance.

### *C. elegans ari1* members genetically interact with germline translational regulators

Studies over the past ~30 years have revealed a complex regulatory network controlling the switch from spermatogenesis to oogenesis during the fourth larval stage of *C. elegans* development^44,45^. Two important regulators of this transition, *fbf-1* and *fbf-2* (the *fbfs*), encode closely related members of the Pumilio and FBF (PUF) family of RNA-binding proteins^46^. *fbf-1 fbf-2* null double mutants fail to initiate oogenesis and show a fully penetrant germline masculinization phenotype as well as a dramatic reduction in the number of germ cells because of their role in maintaining germline stem cell populations^47–49^. In contrast, we and others have shown that partial inhibition of the *fbfs* using an RNAi feeding vector that targets both *fbf-1* and *fbf-2* (sequence similarity prevents targeting of individual *fbf* family members by RNAi) does not generally result in penetrant germline masculinization^50–52^. Notably, a genome-wide RNAi screen for genes that are synthetically lethal with loss of function in *ubc-18*, which encodes a conserved E2 partner of ARI1 proteins in *C. elegans*^25^, identified the *fbfs* among other genes^23^.

**Figure 4:**
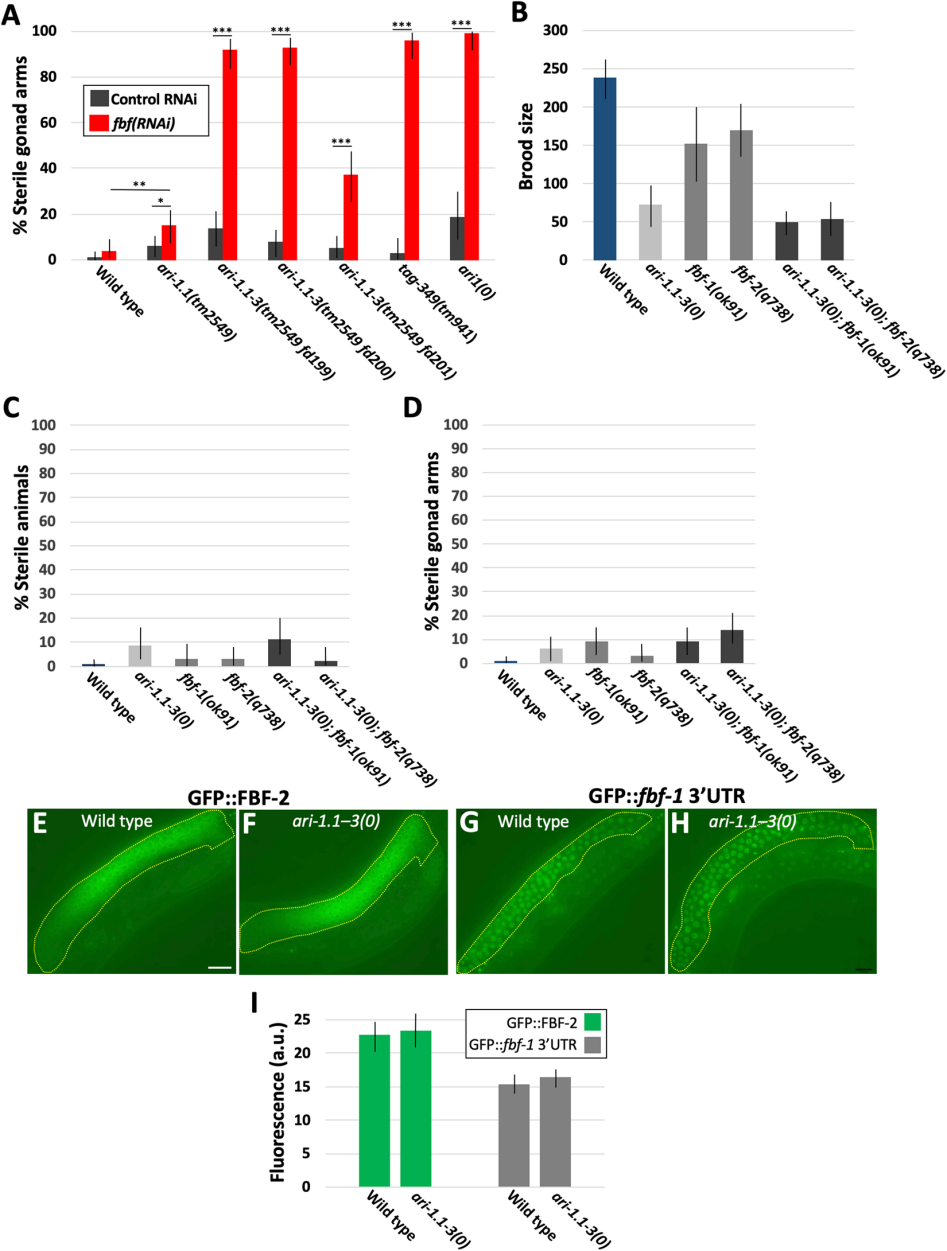
Genetic interactions between *ari1* homologs and the *fbfs*. (**A**) Percentage of sterile gonad arms in wild type and *C. elegans ari1* single and compound mutants following control or *fbf(RNAi)* feeding. *ari1(0)* corresponds to the *ari-1.1–3(tm2549 fd199); tag-349(tm941)* genotype. (**B–D**) Quantification of the indicated genotypes for (**B**) average brood size, (**C**) percentage of sterile animals, and (**D**) percentage of sterile gonad arms. (**E–I**) Analysis of (**E**,**F**) P_*fbf-2*_::GFP::FBF-2::*fbf-2*–3′UTR and (**G**,**H**) P_*pie-1*_::GFP::H2B::*fbf-1*–3′UTR in wild-type and *ari-1.1–3(tm2549 fd199)* strains. Yellow dashed lines indicate regions used to assess expression levels. (**I**) Average levels of the FBF-2::GFP and GFP::*fbf-1*–3′UTR transgenes in arbitrary units. Scale bar in E = 20 µm (**E–H**). Error bars (**A–D,I**) indicate 95% confidence intervals; *p < 0.05, **p < 0.01, ***p < 0.001.

To assay for genetic interactions between the *C. elegans ARI1* genes and the *fbfs*, we performed *fbf(RNAi)* in *ari-1.1(tm2549)*, *ari-1.1–3* deletion, *tag-349(tm941)*, and *ari1(0)* backgrounds. Whereas the F1 progeny of wild-type animals displayed very low levels of masculinization on *fbf(RNAi)* feeding plates, two of the three *ari-1.1–3* deletion strains (*tm2549 fd199* and *tm2549 fd200*) showed >90% masculinization per gonad arm (Fig. 4A). The third deletion strain (*tm2549 fd201*), which also contains a large, incompletely defined insertion, showed significant but somewhat lower levels of masculinization and may thus retain some residual *ari-1.2* or *ari-1.3* activity. These findings are also consistent with our conclusion, based on sequencing data, that *tm2549 fd199* and *tm2549 fd200* represent null alleles of *ari-1.1–3*. In contrast to the triple mutants, the *ari-1.1(tm2549)* single mutant exhibited lower (15%) but statistically significant germline masculinization on *fbf(RNAi)* relative to control RNAi (p = 0.008) and relative to wild type on *fbf(RNAi)* (p = 0.04; Fig. 4A).

Interestingly, *tag-349(tm941)* mutants were strongly hypersensitive to *fbf(RNAi)*, leading to levels of masculinization similar to that of *ari-1.1–3(0); fbf(RNAi)* worms (Fig. 4A). This finding is consistent with our phylogenetic data suggesting that recent gene duplications have likely led to substantial redundancy between genes within the *ari-1.1–3* operon versus *tag-349*, which is present as a single gene locus (Figs 1 and S1). As expected, *ari1(0)* animals grown on *fbf(RNAi)* were uniformly masculinized (Fig. 4A).

**Figure 5:**
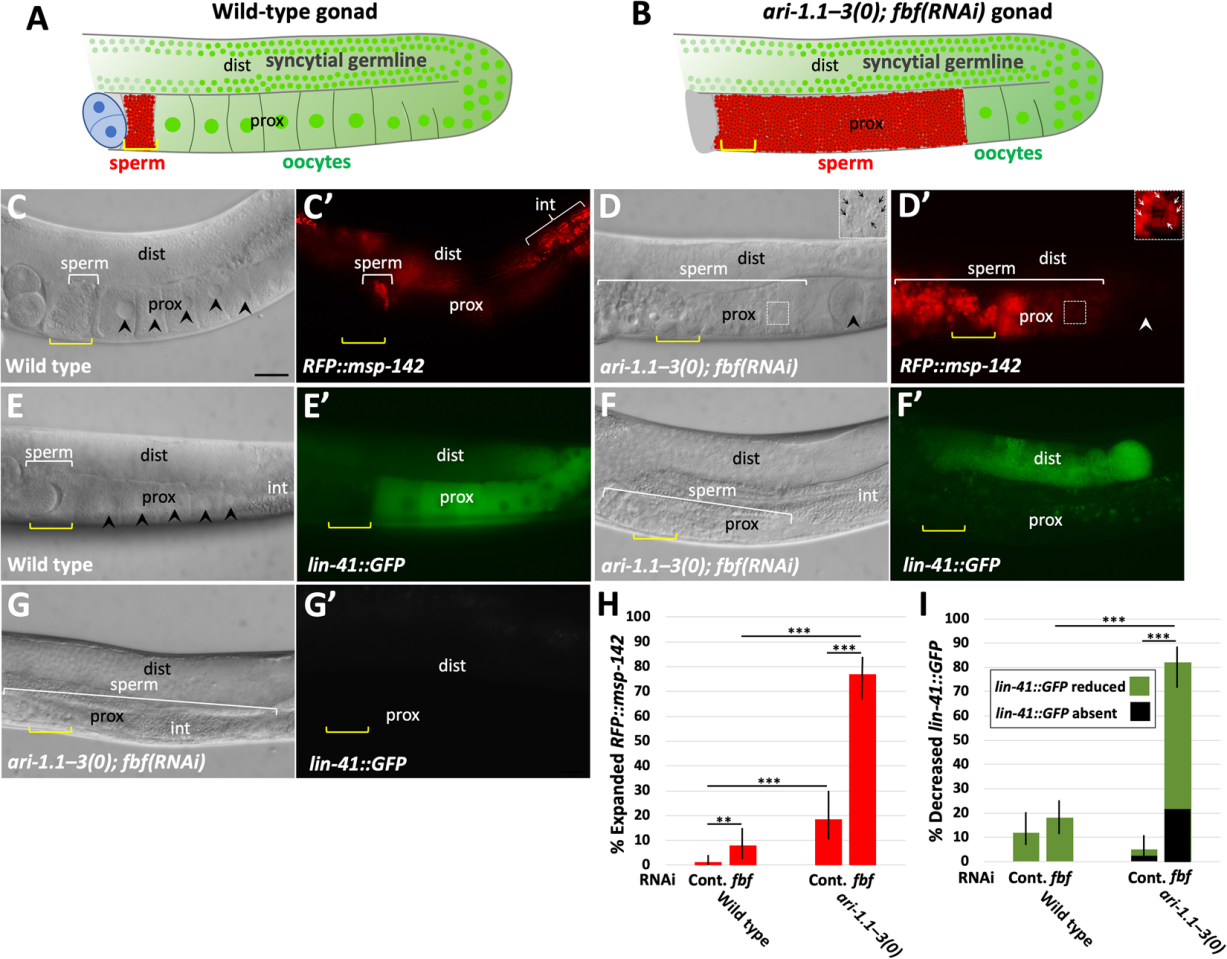
Visualization of germline markers using fluorescent reporters. (**A,B**) Schematics of (**A**) wild-type and (**B**) *ari-1.1–3(tm2549 fd199); fbf(RNAi)* gonad arms, in which red corresponds to the *RFP::msp-142* sperm marker and green to the *lin-41::GFP* oocyte marker. Spermathecae are indicated with yellow brackets (**A–G**); in masculinized gonads, excess sperm are present distal to the spermatheca. Panels A–G are oriented such that ventral is down and the vulva is out of view (lower left side); distal (dist) and proximal (prox) regions of the gonad are also indicated. (**C–G**) Corresponding DIC and fluorescence images of gonads in wild-type and mutant backgrounds. **(C–D**) Representative gonads in (**C**) wild-type and (**D**) *ari-1.1–3(tm2549 fd199); fbf(RNAi)* adult hermaphrodites showing excess *RFP::msp-142* in the mutant including expression distal to the spermatheca and within the uterus (left of spermatheca). Note that intestinal fluorescence (int) in Panel C’ is due to gut granule autofluorescence. In addition, the fluorescence intensity of the inset region in D’ was boosted relative to the main panel for improved visibility. (**E–G**) Representative gonads in (**E**) wild-type and (**F,G**) *ari-1.1–3(tm2549 fd199); fbf(RNAi)* adult hermaphrodites showing a proximal reduction (**F**) or absence (**G**) of *lin-41::GFP* in mutants. (**H,I**) Quantification of phenotypes shown in (**C**–**G**). Error bars indicate 95% confidence intervals; **p < 0.01, ***p < 0.001. Scale bar in C = 20 µm (**C–G**).

To extend our observations on germline masculinization, we visualized sperm in wild-type and *ari-1.1–3(0); fbf(RNAi)* backgrounds using a reporter for the major sperm protein *msp-142*^53^. Consistent with DIC observations, expanded (distal) expression of a *msp-142::RFP* reporter was observed in ~75% (n = 96) of gonad arms in *ari-1.1–3(0); fbf(RNAi)* hermaphrodites (Fig. 5A–D,H). In addition, we observed an increased frequency of *msp-142::RFP* expansion in *ari-1.1–3(0)* strains relative to wild type on control RNAi plates (Fig. 5H), consistent with DIC results for *ari-1.1–3(0) mutants* on NGM plates (Fig. 3B–D). Correspondingly, expression of a reporter for oogenesis (*GFP::lin-41*)^54^ was reduced or absent in ~80% (n = 100) of *ari-1.1–3(0); fbf(RNAi)* gonad arms versus ~15% in wild-type animals treated with *fbf(RNAi)* (Fig. 5A,B,E–G,I). We also observed that *ari-1.1–3(0); fbf(RNAi)* gonads that expressed *GFP::lin-41* in the distal portion of the gonad only (i.e., not in the proximal region), typically failed to produce mature oocytes.

Our findings using *fbf(RNAi)* could be attributable to functional interactions between the *ari-1* homologs and *fbf-1*, *fbf-2*, or both *fbfs*. To test this, we generated strains containing *ari-1.1–3(0)* together with deletions in either *fbf-1* (*ok91*) or *fbf-2* (*q738*). Both *ari-1.1–3(0); fbf-1(ok91)* and *ari-1.1–3(0); fbf-2(q738)* strains were viable as homozygotes and had brood sizes similar to *ari-1.1–3(0)* strains (Fig. 4B). In addition, no evidence for strong enhancement of sterility was observed in the compound mutants relative to *ari-1.1–3(0)* (Fig. 4C,D). Our findings indicate that the potent genetic interactions detected between *ari1* family members and the *fbfs* are not attributable to specific interactions with either *fbf-1* or *fbf-2* but require partial inactivation of both *fbf* family members. This finding is analogous to results obtained for genetic interactions between the *fbfs* and *fshr-1*^50^, a FSHR-like receptor, but contrasts with other studies reporting genetic interactions specific to either *fbf-1* or *fbf-2*^55,56^.

One explanation for the hypersensitivity of *ari1* mutant strains to *fbf(RNAi)* is that the ARI1s might positively regulate FBF expression or activity. If so we would expect to see reduced or altered expression of the FBFs in *ari1* mutant strains. To test this, we made use of a CRISPR-generated GFP::FBF-2 reporter that expresses FBF-2 under the control of the endogenous *fbf-2* promoter and 3′UTR regulatory sequences (gift of G. Seydoux). We chose to examine a GFP::FBF-2 expression in the *ari-1.1–3(0)* background as these strains are healthier than *ari1(0)* mutants but nevertheless show dramatic enhancement of germline masculinization with *fbf(RNAi)*. Our analysis did not detect any qualitative or quantitative differences in the pattern or intensity of the GFP::FBF-2 reporter in wild-type and *ari-1.1–3(0)* animals (p > 0.05, n ≥ 21 for each genotype), indicating that ARI-1.1–3 are unlikely to regulate FBF-2 levels (Fig. 4E,F,I). We note that we were unable to examine GFP::FBF-1 levels in *ari-1.1–3(0)* mutants because of variable germline silencing of the available transgene^57^. However, previous studies have shown that FBF-1 and FBF-2 are mutually repressive, such that loss of *fbf-1* leads to substantially increased FBF-2 levels and loss of *fbf-2* leads to a corresponding increase in FBF-1^48^. Thus, our observation that GFP::FBF-2 levels did not vary between wild type and *ari-1.1–3(0)* also implies that FBF-1 activity is unlikely to be strongly altered in these mutants.

Given that ubiquitination can potentially affect protein activity without changes in abundance, we also examined expression of a *P*_*pie-1*_*::GFP::H2B::fbf-1–*3′*UTR* reporter that expresses nuclear-localized GFP in the germline under the control of *fbf-1* 3′UTR sequences^57^. Because FBF-2 regulates *fbf-1* through its 3′UTR, this reporter provides a convenient readout for FBF-2 activity. Similar or identical expression patterns and levels of expression were observed for the *fbf-1–*3′*UTR* reporter in wild-type and *ari-1.1–3(0)* strains (p = 0.15; n ≥ 19 for each genotype; Fig. 4G–I), consistent with FBF-2 activity being unaltered in *ari-1.1–3(0)* mutants.

Taken together, our results indicate that *C. elegans* ARI1s collectively promote the switch from spermatogenesis to oogenesis during development and may function in parallel to or downstream of the FBFs. However, the viability of *ari1(0)* homozygotes indicates that they are not essential for this process. Rather, this function is revealed most clearly under conditions in which oogenesis is partially compromised, such as when function of the FBFs is reduced.

### UBC-18, but not UBC-3, promotes the switch to oogenesis

**Figure 6:**
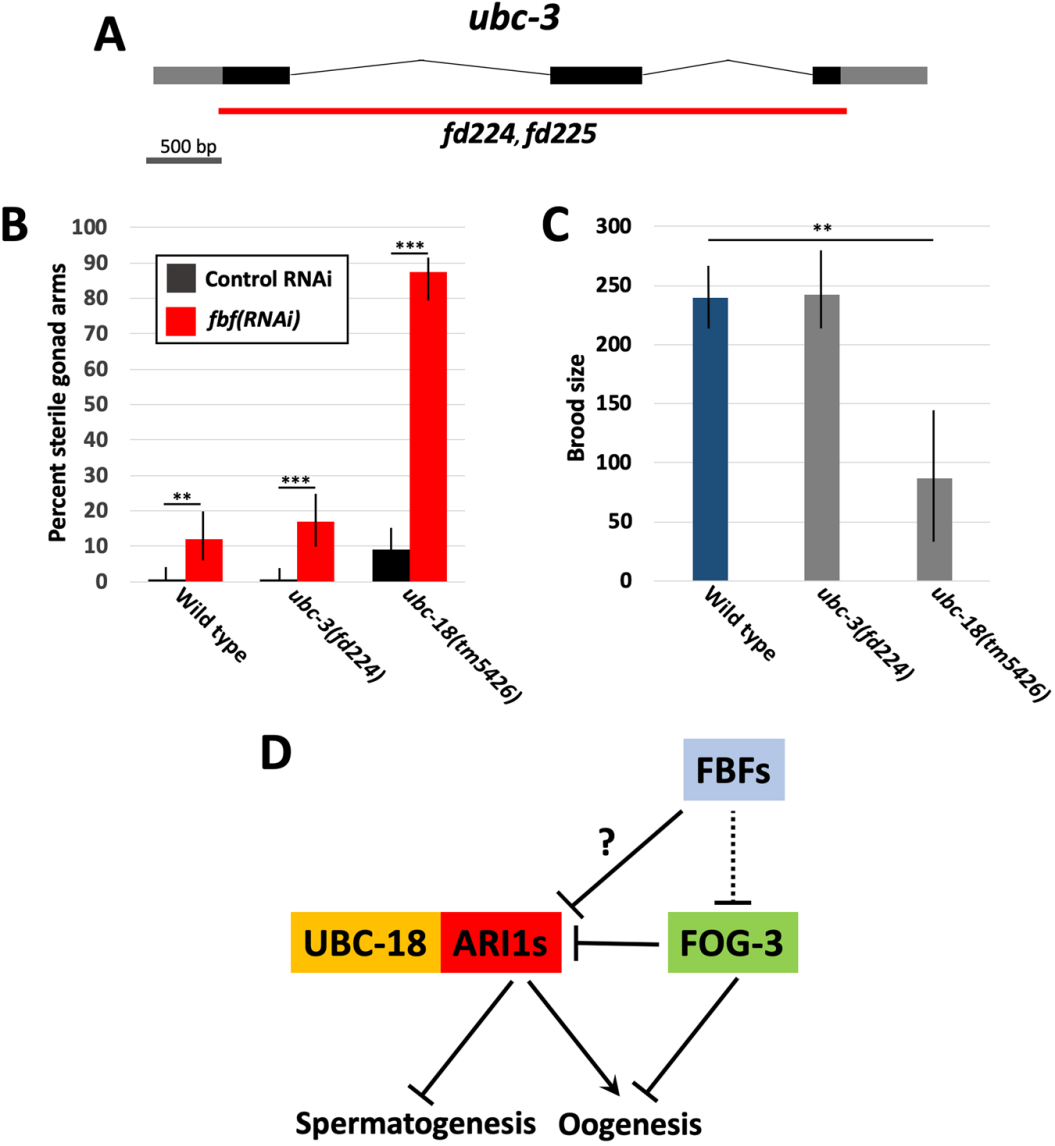
Analysis of *ubc-3* and *ubc-18* mutations. (**A**) Gene diagram of *ubc-3* showing the extent of deletion mutations (red line). (**B**) Percentage of sterile gonad arms in wild type and mutant strains following control RNAi or *fbf(RNAi)* feeding. (**C**) Average brood sizes of wild type and mutant strains. (**B,C**) Error bars indicate 95% confidence intervals; **p < 0.01, ***p < 0.001. (**D**) Model based on published findings (see text) and our results for how ARI1–UBC-18 regulates germline development in conjunction with several RNA binding factors.

ARI-1.1 partners directly with the E2 ubiquitin-conjugating enzyme UBC-18 to control pharyngeal development^23,25^. In addition, in conjunction with an SCF-like complex, ARI-1.1 can partner with the E2 UBC-3/Cdc34^23^ to mediate the poly-ubiquitination of targets. Collaboration among multiple E2s and E3s to achieve substrate poly-ubiquitination may be a unique biochemical property of HHARI family members, although the extent to which this occurs is unknown. We therefore sought to test if *ubc-18* and *ubc-3* mutants were also defective in the switch to oogenesis based on hypersensitivity to *fbf(RNAi)*. Because there were no available characterized mutations in *ubc-3*, we used CRISPR/Cas9 to generate *ubc-3* deletion strains. *ubc-3(fd224)* contains a 2233-bp deletion (LGI 1698512–1700746), whereas *ubc-3(fd225)* contains a 2232-bp deletion (LGI 1698509–1700742) (Fig. 6A). Both alleles are homozygous viable and are likely to represent null alleles of *ubc-3*.

As shown in Fig. 6B, *fbf(RNAi)* induced high levels of sterility and masculinization in *ubc-18(tm5426)*, similar to results for *ari-1.1–3(0)* mutants (Figs 4A and 6B). This finding suggests that the ARI1s partner with UBC-18 in the regulation of germline differentiation as well as pharyngeal development. Consistent with this, *ubc-18* is expressed in the germline^27–29,58^, and *ubc-18(0)* mutants have strongly reduced brood sizes relative to wild type (Fig. 6C)^34^. In contrast, *ubc-3* mutants were not hypersensitive to partial knockdown of the *fbfs* and had brood sizes similar to those observed for wild type (Fig. 6B,C, and data not shown). These findings indicate that UBC-3 is unlikely to function in collaboration with the ARI1s in the regulation of germline differentiation.

## Discussion

In summary, our data are consistent with the *C. elegans* ARI1 family members acting with UBC-18 to promote the posttranslational modification of targets controlling germline differentiation. This could occur through the ARI1s inhibiting spermatogenesis, promoting oogenesis, or both (Fig. 6D). It is tempting to speculate that the ARIs may promote the degradation of one or more pro-spermatogenesis proteins, thereby facilitating the transition to oogenesis. Indeed, ubiquitin-mediated proteolysis has been implicated in the regulation of *C. elegans* sex-specific differentiation^59–66^. However, HHARI members specifically promote mono-ubiquitination^18,19^, raising the possibility of non-degradative regulation of germline targets by the *C. elegans* ARI1s^3,67^.

Consistent with a pro-oogenesis function, all four *C. elegans* ARI1s were recently identified as targets of FOG-3, an RNA-binding protein and germline translational repressor that promotes spermatogenesis^68^ (Fig. 6D). Notably, 94% of FOG-3 targets are pro-oogenic mRNAs, consistent with our experimental findings. Furthermore, an analysis of mRNA transcripts from dissected feminized versus masculinized gonads suggests that the ARI1s are expressed at higher levels in oogenic gonads^69^. Additionally, screens for FBF-binding/regulatory sites in *C. elegans* identified regions within several *C. elegans* ARI1 family members^70,71^, suggesting that that the ARI1s and FBFs could be directly linked within the germline regulatory network (Fig. 6D). Taken together, these studies indicate that the *C. elegans* HHARI homologs promote oogenesis and may be regulated by several well-characterized germline RNA-binding proteins (Fig. 6D).

A *D. melanogaster ariadne* member, *ari1*, is prominently expressed in fly ovaries including germline nurse cells^32^. In addition, although *ari1* is not essential for oogenesis, certain alleles of *ari1* are deleterious to normal oogenesis, suggesting that pro-oogenic functions for ARI1 family members may be conserved. It is also worth noting that rapidly evolving genes in genera including *Drosophila* and *Caenorhabditis* appear to show a bias for expression in sperm or oocytes, consistent with our phylogenetic observations^72–76^.

In addition to germline defects, we noticed that *ari1(0)* animals displayed sluggish movement on Petri dishes as well as in liquid medium (D.S.F. and J.A.P., unpublished findings), suggesting additional functions within muscle cells or neurons, tissues in which *ari1* family members are also expressed^25,39^. These observations implicate *C. elegans* ARI1 proteins in developmental and/or physiological functions outside the germline, consistent with published findings in *Drosophila* and mammals^32,77–81^. It will ultimately be of interest to identify the specific targets of the *C. elegans* ARI1s, as relatively little is currently known about the physiological substrates of ARI1 family members^15^.

## Methods

### Phylogenetic analysis

Orthologs for ARI1 and ARI2 genes included in our study were identified by reciprocal BLAST homology. Amino acid sequences were aligned using MAFFT v7.294^82^ with the L-INS-I alignment strategy. Maximum likelihood phylogenetic tree reconstruction was carried out using RAxML v8.2.8 with 1000 bootstrap replicates and the flatworm model *Schmidtea mediterranea* included as the outgroup. The species tree included for reference in this paper was generated by assignment of genes from the complete genomes to orthogroups and subsequent alignment and tree construction with Orthofinder v2.2.5, Stride, and STAG, respectively^83–85^. Whole-genome protein sequences for the flatworms and roundworms were obtained from WormBase^86^; sequences for human, fruit fly, and mouse were obtained from NCBI. Both trees were prepared for publication using FigTree v1.4 (http://tree.bio.ed.ac.uk/software/figtree/) and then manually edited for clarity.

### *C. elegans* strains

The following strains were used: WY683 [*ari-1.1(tm2549)*], WY1326 [*ari-1.1(tm2549) ari-1.2/3(fd199)*], WY1327 [*ari-1(tm2549) ari-1.2/3(fd200)*], WY1328 [*ari-1(tm2549) ari-1.2/3(fd201)*], WY1488 [*tag-349(tm941*)], WY1491 [*ari-1(tm2549) ari-1.2/3(fd199); tag-349(tm941)*], WY1413 [*ubc-3(fd224)*], WY1414 [*ubc-3(fd225)*], WY1334 [*ubc-18(tm5426)*], DG5913 [*lin-41::GFP*], WY1365 [*ari-1(tm2549) ari-2/3(fd199); lin-41::GFP*], DG5897 [*msp-142*::RFP], WY1372 [*ari-1(tm2549) ari-2/3(fd199); msp-142::RFP*], WY1416 [*ari-1(tm2549) ari-1.2/3(fd199); fbf-2(q738)*], WY1459 [*ari-1(tm2549) ari-1.2/3(fd199); fbf-1(ok91)*], JH3201 [*GFP::fbf-2(axIs2057)*], JH3201 [*GFP::fbf-2(axIs2057)*], JH2270 [*unc-119(ed3); axIs1654* [*pie-1p::GFP*::histone H2B::*fbf-1* 3′*UTR* + *unc-119*(+)], WY1397 [*ari-1(tm2549) ari-2/3(fd199); axIs2057*], and WY1493 [*ari-1.1(tm2549) ari-1.2/3(fd199); axIs1654*].

### CRISPR/Cas9 editing

Deletion of *ari-1.2*/C27A12.2 and *ari-1.3*/C27A12.6 was carried out using standard plasmid-based co-CRISPR methods^87,88^. sgRNA-expressing constructs were generated using the Q5 Site-Directed Mutagenesis kit (E05545, NEB) and plasmid pDD162 (Peft-3::Cas9 + empty sgRNA), a gift from Bob Goldstein (Addgene plasmid # 47549)^89^. The sgRNAs targeted sequences flanking C27A12.6 (pKRD12, 5′-agatcattgcgctgaaggtg-3′; pKRD1, 5′-aatgaatacccttaatgagc-3′; pKRD2, 5′-ttcttactccggctcattaa-3′) and C27A12.7 (pKRD14, 5′-tggtgtccaggggttgattg-3′; pKRD15, 5′-gaagtaccgtaaaatgatgg-3′; pKRD13, 5′-cctcgacaacgacttgttgg-3′). After injection of the individual sgRNA constructs into worms and isolation of resulting strains**]**, PCR screening using primers 5′-tagcacacacacaccgttca-3′ and 5′-agtggtgccccggtatagat-3′ was carried out to identify strains containing deletions. Sequencing of WY1326 revealed a 3791-bp deletion (linkage group I (LGI) 6054173–6057963) along with a 25-bp insertion after bp 6054173 (ctcttaagca–TCTTCGAGGTCCTAAAAGAAGAAAG–gtagaacaac). Sequencing of WY1327 revealed a 3988-bp deletion (LGI 6053993–6057980) containing a large insertion that was not fully characterized. Sequencing of WY1328 revealed a 3850-bp deletion (LGI 60541186057967) with a 4-bp insertion after bp 4054118 (ggtcctcgac–CTTT–gacatttggc).

Deletion of *ubc-3* was carried out using standard preassembled Cas9/gRNA Ribonucleoproteins. (RNPs) together with co-CRISPR methods^88,90^ using the *dpy-10* dominant marker and guide sequences targeting *ubc-3* (5′-gtagagcgtcttcgg-3′ and 5′-gtagagcgtcttcgg-3′). PCR screening was carried out with *ubc-3*−specific primers 5′-ttgcaaccaggagaagacgg-3′ and 5′-gaaagtgcactgtgatcagcc-3′. Sequencing identified *ubc-3(fd224)*, which contains a 2233-bp deletion (LGI 1698512–1700746; gacgctctac–tataatgatg) and *ubc-3(fd225)*, which contains a 2232-bp deletion (LGI 1698509–1700742; gaagacgctc–ggactataat).

### Phenotypic analysis

Strains were propagated at 25 °C (JH2270 and WY1493) or 22 °C (all others) using standard methods^91^. RNAi feeding was carried out using established methods^92^. Brood size for wild type was determined with n = 5; brood sizes for mutant strains were determined using n = 9–16 (Table S1). L4-stage larvae were placed on NGM plates and moved to new plates each day for a minimum of 6 days, and all progeny were counted. Percentage of sterile animals was determined using n = 88–100; percentage of sterility gonad arms, n = 78–100. Microscopy was carried out using a standard epifluorescence microscope (Nikon E600) with DIC and filters for GFP and RFP. Sterile animals were identified by the absence of any embryos within the uterus. Sterile gonads were determined by examination of gonad arms and by the stages and positions of embryos within the uterus. Worms with two fertile gonad arms contain early-stage embryos adjacent to both the anterior and posterior spermathecae, with an ordered progression to more late-stage embryos moving proximally towards the vulva. In contrast, worms with just one fertile gonad arm show developmental progression from the fertile gonad only, with late-stage embryos typically found adjacent to the spermatheca of the sterile gonad. *RFP::msp-42* expression (Fig. 5H) was scored as having expanded if the width of the RFP-positive region adjacent to the spermatheca was increased >2-fold relative to that of wild type. *lin-41::GFP* expression (Fig. 5I) was scored as having decreased if expression was missing in the region normally occupied by proximal oocytes. Expression levels of FBF-1 and FBF-2 markers (Fig. 4) were carried out in mid-stage L4 hermaphrodites (as determined by vulval development) using ImageJ quantification software with background subtraction. Statistical comparisons of means were calculated using a two-tailed Student’s t-test. Statistical comparisons of proportions were calculated using a Fischer’s exact test.

## Electronic supplementary material

Supplementary Information
