# Supplementary material for: Regulation of germ cell development by ARI1 family ubiquitin ligases in C. elegans
Source: Sci Rep. 2018 Dec 10;8:17737. doi: 10.1038/s41598-018-35691-y (PMC6288150; doi:10.1038/s41598-018-35691-y)
Supplement: Supplementary file 1 — Supplementary Information [file 41598_2018_35691_MOESM1_ESM.pdf]

Supplemental Information:

Regulation of germ cell development by ARI1 family ubiquitin ligases in *C. elegans*

Julian A. Poush[1], Nicolas A. Blouin[1,2], Kristin R. Di Bona[1], Vladimir Lažetić[1], and David S. Fay[1]*
[1]Department of Molecular Biology, College of Agriculture and Natural Resources, University of Wyoming, Laramie, WY 82071; [2]Wyoming INBRE Bioinformatics Core

Contents:

Figure S1. Ariadne family members.
Table S1. Supplemental data on brood sizes
File S1. Table of amino acid sequences used to generate the summary gene tree in Figure S1.

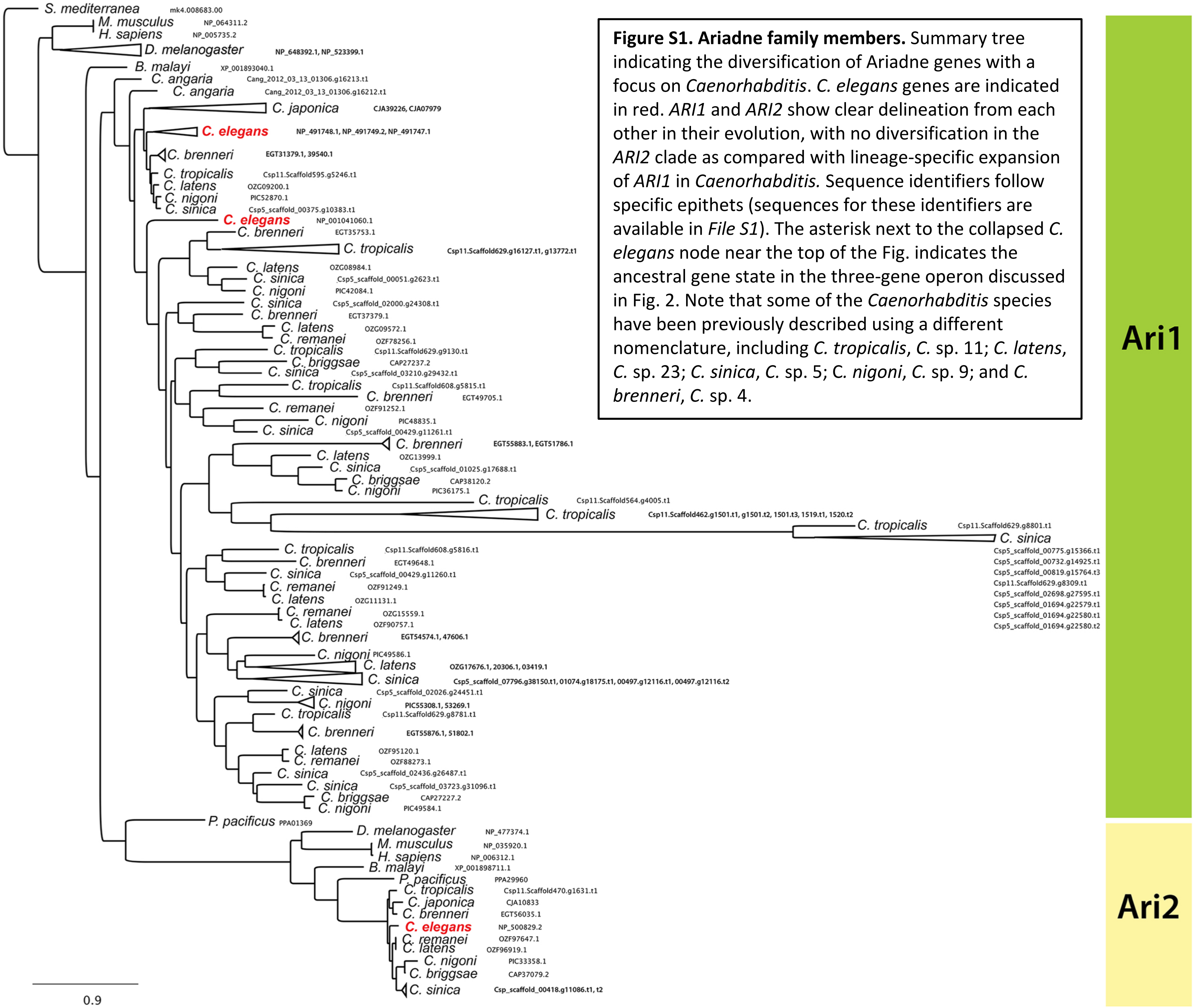

Figure S1. Ariadne family members. Summary tree indicating the diversification of Ariadne genes with a focus on *Caenorhabditis*. *C. elegans* genes are indicated in red. *ARI1* and *ARI2* show clear delineation from each other in their evolution, with no diversification in the *ARI2* clade as compared with lineage-specific expansion of *ARI1* in *Caenorhabditis.* Sequence identifiers follow specific epithets (sequences for these identifiers are available in *File S1*). The asterisk next to the collapsed *C. elegans* node near the top of the Fig. indicates the ancestral gene state in the three-gene operon discussed in Fig. 2. Note that some of the *Caenorhabditis* species have been previously described using a different nomenclature, including *C. tropicalis*, *C.* sp. 11; *C. latens*, *C.* sp. 23; *C. sinica*, *C.* sp. 5; C. *nigoni*, *C.* sp. 9; and *C. brenneri*, *C.* sp. 4.

Table S1. Supplemental data on brood sizes

Genotype	Average brood size	Standard deviation	Coefficient of variation (%)	Range	N
Wild type	239.2	30.4	12.7	198–275	5
ari-1.1(tm2549)	184.7	42.2	22.9	107–233	9
ari-1.1-3(tm2549 fd199)	72.1	52.8	73.2	0–144	16
ari-1.1-3(tm2549 fd200)	70.1	31.7	45.2	28–120	10
ari-1.1-3(tm2549 fd201)	72.7	23.1	31.8	53–134	10
tag-349(tm941)	286.7	52.7	18.4	195–386	13
ari(0) (tm2549 fd199; tm941)*	44.3	43.8	98.9	0–180	18
fbf-1(ok91)	152.2	102.8	67.5	0–339	17
fbf-2(q738)	169.7	82.3	48.4	8–299	19
ari-1.1-3(tm2549 fd199); fbf-1(ok91)	50.2	34.4	68.5	0–116	15
ari-1.1-3(tm2549 fd199); fbf-2(q738)	53.4	35.1	65.7	0–118	14

* *ari(0)* indicates *ari-1.1-3(tm2549 fd200); tag-349(tm941)*

File S1. Table of amino acid sequences used to generate the summary gene tree in Supplementary Fig. 1.

Taxon GenBank or WormBase gene ID Amino Acid Sequence
Brugia malayi XP_001893040.1
MSLGSDEAMDDDGAFSDGSESDATSTYSDDDGIVVDPPVIKTERGGSHDSESADFQVLDAEKVTFEMNKI
IEDVASVLRLSATICRLLLHHYKWNKESLLERFYESTDMDSFFLDANIISPFKVARRGDEGLADIVDTCV
ICCNRTILTGLQCSHRFCYPCWDSYLTTKIMEEGRAHVACPQHNCPIIVDDEKTLALVKSENAKKRYRRL
IINSFVECNRLLRWCPAADCGRVIEVGHLEARPVKCTCGTVFCFACGHEWHEPVNCRLLKLWIKKCNDDS
ETSNWISANTKECPKCQVTIEKDGGCNHMTCKNVACKMEFCWMCLGPWEPHGSSWYSCNRYDDTLAKQAR
DAQERSRAALQRYLHYYNRYMNHQQSLKLEHKLYSIVKSKMEVMQQANMSWIEVQFLRKAVDVLSECRRT
LMYTYAFAFYLQKDNQSVIFEENQRDLEHATEQLSEFLERDLDHENLVSLKQKVQDKYRYVEQRRTTLLK
HCAEGVEHDFWRFTA
Brugia malayi XP_001898711.1
MECGDAPADSPSFSDDEADYDYDDFYCNGENCGMEIFEREDPEYIEYACLKVPEVERLLKETVDHVVSTL
NVSSSLAKILLHFYKWDDSTLIQLYRVDPCKVLVDCFVCAGSSKQQPDTMSCVVCTRLQDECTKMYALDC
GHSFCSACWMEYIETQLCNGLSITIGCMASGCTLLCLEDFVLRILSERTEIRDKYERLIFKDCVESHPQL
RFCPGIDCHVVIKAQCQKAKKVTCTSCRISFCFQCGCDYHAPTSCETIRKWLTKCADDSETANYISAHTK
DCPNCHSCIEKNGGCNHMQCAKCKYHFCWMCFGDWKNHGSEYYECSRYKENPSIAQEANHVKARRALEKY
LHYYERYENHHKSLKMEENLRNCIMKKIDEKVNGYEGTWIDWQYLHRAATLLTKCRYTLQYTYPYAYYME
NGPRKQLFEYQQAQLEKEIEELSWKVERAESTERGDLETQMHVAECKRRTLLQDFFD
Caenorhabditis angaria Cang_2012_03_13_01306.g16212.t1
FSRFYEHPDTNAFLLDAQVIPNVTESSSSAQPNPAECDICCVVCELSGLACNHRSCARCWKAYLQTKIMN
DCSSEIECMCSGCKLLIEDEKVMNYITDEVTRAAYRRLVISSYIETNRLLRWCPGVDCGKAVKVPHCEPR
LVICSCSKQFCFACGNDWHEPVNCRLLKLWLKKCSDDSETSNWINANTKECPKCQITIEKDGGCNHMTCK
SSTCKFEFCWMCLGPWEPHGSSWYSCNRFDDSAAKQARDAQELSRANLQRYLFYYNRYMGHQQSLKLEGK
VC
Caenorhabditis angaria Cang_2012_03_13_01306.g16213.t1
MQQRSMSWIEVQFLRKAVDVLSECRRTLMFTYAFAYYLQKDNNSQIFEDNQKDLEMETEKLSEILERELE
NENLVMLKQKVQDKYRYVEHRRKILLSHCQEGTERDQWKYIE
Caenorhabditis brenneri EGT31379.1
MSDDEINMDDSAESDQGEEDECLSEDDGIALESHDPNASEYRENAEPDNEVLNHDQLEAEMKKTIADVQA
VLQVKSGTCRILLHKYKWNKESLLERFYEHPDTNAFLIDAQVIPRHTEKLPCGESECDICCMVSELSGLA
CNHRACTPCWRSYLTNKILDGAQSEIECMAANCKLLIEDEKVMFYITDPAIIASYRRLIVASYVETNRLL
KWCPGVDCGKAVRVNHCEPRLVVCSCGSRFCFSCGNDWHEPVNCRLLKLWMKKCSDDSETSNWINANTKE
CPKCMITIEKDGGCNHMTCKNTTCRFEFCWMCLGPWEPHGSSWYNCNRFDDTVAKTARDAQELSRANLQR
YLFYYNRYMGHQQSLRLEGKLYATVKSKMEQMQTLSMSWIEVQFLRKAVDILSECRRTLMFTYAFAFYLR
RDNNSIIFETNQKDLEMETEQLSGFLERDLETENLVTLKQKVQDKYRYVEHRRRILLDHCSEGAEQDLWQ
FNE
Caenorhabditis brenneri EGT35753.1
MSSDEEEMYMEADDFTEATQEDEFLDEEDLEEDMKQSIESVHSVLQVSNGMCRLLLQKYKWSKDALLDRF
YENPDPVSFLIDAHILPSQSVTNGSGDTAPPTECQICCMDGEELSGLACNHLACNDCWKCYLQSKIKEGQ
SEIQCMASDCKLLLEDETVLKYIKDADSYRKVLVNSYVETNKMLRWCPGKNCGKAVKIAGLDRNMIICPC
GSRFCFTCGLEGHEPINCRLLKLWIKRCQDDSETYNWINANTKDCPKCNAPIEKNGGCNYMRCQNTSCKF
EFCWLCFGSWKDEGAHNCNRFDEKKDGKGRDQARISLEKYLFYYNRYINHLKSLQLERKLKDLVAEKMES
MQEMTMSWVEVQFLDKAVSVLSECRRTLMYTYAFAFYLQRDNNSIIFEANQKDLETSTEQLSHLLERELD
FEDLVVLKQKVQDKYRYVEQRRKVLLDHCAEGKDLDFWVFNE
Caenorhabditis brenneri EGT37379.1
MYYDDDSVYENDDDFEKEEVQDQLLLPDDLESEMADLILDVRSVLQVSEGMARILLHKFKWNKNSLLDKF
YESPDTEAFLVEAQVIPKTTQPSSTGEDDCDICCSFGELTGLACNHRACEDCWKHYLTEKIMEGGSSEIE

CMSPDCKLLIEDEKIKFYIRDKTILDKLQRLVINSFVETNPVLKWCPGQNCQKAVKVADPEPRLISCPCG
TQFCFSCCQNWHAPADCALLKKWLKKCMDDSETCNWINANTKECPKCFVPIEKNGGCNHMRCTNKSCKFE
FCWMCMGCWRSHGTAGYQCNRFNEGQDANRSKHRAYLNRYLFYYNRYITHQQSLALEEKLKETVAAKMAE
MELKGMAWIEVQFLKKSIDALAKCRRTLMYTYVFAFYLKKSNHSEMFENNQRDLEMATEQLSGFLERDLE
NEDLVTLKQKVQDLTKYVDRRQEALLEHCADGNEQDLWEFTENGV
Caenorhabditis brenneri EGT39540.1
MSDDEINMDDSAESDQGEEDEPELAQYGPNGSEGSKRPESCLSEDDGIALESHDPNASEYRENAEPDNEV
LNHDQLEAEMKKTIADVQAVLQVKSGTCRILLHKYKWNKESLLERFYEHPDTNAFLIDAQVIPRHTEKLP
CGESECDICCMVSELSGLACNHRACTPCWRSYLTNKILDGAQSEIECMAANCKLLIEDEKVWKVMFYITD
PAIIASYRRLIVASYVETNRLLKWCPGVDCGKAVRVNHCEPRLVVCSCGSRFCFSCGNDWHEPVNCRLLK
LWMKKCSDDSETSNWINANTKECPKCMITIEKDGGCNHMTCKNTTCRFEFCWMCLGPWEPHGSSWYNCNR
LVSD
Caenorhabditis brenneri EGT47606.1
MSSDEEDYGEESEYETDEIEMDDDSDKFLTPSDLTAEMNLAITDAQSILQVNAGVCRLLLQKFKWNKNSL
LDKFYENPDTNEFLTKSNIIPCKLEPIGSQSDECEICCMESELVGLQCNHLACQECWTHYLSERVKANQS
EIECMTTDCKLLIPDEQIKKFICDENLKNSFDRVTINNYVEANPYLTWCPADYCSKAVKVVNTGTRLITC
PCGTIFCFTCGNDGHDPVSCRHLKLWQKKCEEVKSKATTGDGYSSDNDTFKWILSNTKDCPQCLTAIEKN
GGCNRILCRNKKCMFEFCWLCMKSWAIHGYSACNIFTEEAEKKRVDSRAELLRLQFYLNRFMEHDRSLQL
EKKLVRTVETQMEKIQDLTKSWTETQFLRKAVDVLSECRRTLKFTYAFAYYLERNNHALLFETNQKDLEM
AVEQLSGFLEKDLENDDVATLKQKVQDKSRYVEHRRKVLVDHCFEGNEQEFWSFRE
Caenorhabditis brenneri EGT49648.1
MDFSDGEPEDFDVDDVIDIDEVEEEVQETSYEVLDATDIETQLNEAISDLQDVLQVTRGVSRILLQKFKW
NKDELLEKFYEKPDTEAFLVEAQVLPKEPAPTLPMTPEDECEICCDSAPLSGLACGHKACDMCWGTYLAD
KIKEGQSEIQCMASDCKLLMEDVKIQSYINDPSLISKYHQLIIRSYVETNKLLSWCPGMNCGKVVKVHYS
ESRLVVCSCGTQFCFMCGSKAHDPVSCRLLKLWKKKTEELHGKKHATEGYGADDDSFKWLMTNTKDCPKC
MVPIEKNGGCNYMLCKNSKCRFQFCWVCMQPWQVHSQAWYECNKYDPAAAVSREKKRAEHHRLIFYYTRY
MAHEQSLAFEAKLRRMVRLKVLRMEQLLIPWIDAQYLFKAVDTLVKCRNTMMFSYVFAYFLKRDNNSLIF
EANQRDLEKATEELSGFLERDLEKQDYTKLKQMVNDKSSYVDKRRDVLLKHLRDGEEMGVWEFNE
Caenorhabditis brenneri EGT49705.1
MALDDYVEEIYMEKEKEKRQEKAKPLYDLLTQEQLKSDMDNIITDAQSVLQVPRGICRLLLQAYNWNKDT
LFDHFYESPDTATFLNTVHIGLPDPGIVFTMGECDVCFEMGELSGPSCSHKACAGCWKGYLEDKIRSDGV
CDMNCMMPNCELLLEDEKVLFYITDPALISLYHKLTVNNYVSSNRRLKWCPGIDCGRAVKLPDTSRHFVS
CPCGAEFCSSCGHDFHEPLTCEMMKKWNSKTQESSKTLVWIARFTKPCPKCSTPIEKDGGCNVIVCTLPT
CRLQFCWICMRPFDKDHMQFCSYYDVSQNTGDEATEDRVNVARHLFYHDRYIGHRQSLEFERKLRKIVDS
NIEYLRLDCHASGQLKTAMEALFASRRTLMNSYIFAFFLQKDNNARIFENNQADLHGAVEKLSKTLHDKI
KKQRPENLGKLLTEIHDKCVYVEHRRKILLDHCKEGYDYNFWKFEEQPF
Caenorhabditis brenneri EGT51786.1
MDIRVTSADEEENTFDTNWNDLPPEVKTRCIQKMENADRLHLRQTSKTEKYLVDVEGIGLEKKKRGDQKV
ENLVEKDEDAAGEYCPIYGESDDDDDKDEPDFKVRNHESLYTEMKKKIREAQELLEMRPGICQILLQKYK
WSVAFLMEKFYDNPDRGAFLTAVNVDPSEHLHAVVGECQICFEEQELTGLACEHRYCWDCLREYMIDKIF
DGQSEIKCIGLECPLVFEEEKIGSIVIDPEVMSCYHRLLVQKYVQNDAFMKSCPDLSCENTIQVLNPSIR
HVKCNCGYSFCFSCGNDSHEPISCRYLDKWLLKGPEDQSSVWILTNTKKCPRCNAPIEKNGGCMHMTCHS
KDCRYEFCWLCMRDWRAHANCNDFQRTNDAAREAMINKTIADRDRYKFYHDRYAGHMQSLKLEKPLRAKF
DEQMKEIEEDGDRDLRDFQYIYVAIDALSASRRTLMHSYVFAFFLEENYSAIIFKSNQADLNDATENLSK
VLEEFVNSESVGFAEDSKKEIMHKSQYVEQRRKALIEHCIEGDDNDDWVFNE
Caenorhabditis brenneri EGT51802.1
MSSDEAENLYSDGSEAQEEIEVDDYKILDTASIESTMKKQIGEIEILLGVSEGVGRLLLQAHKWNKDSIT
DKFYDSPDRDTFLIESNIIPTDPQPFEEGEAECEICCETTELVGLDCNHRSCKECWKAYLTEKIKDGQSE
IECMDSKCKLLLKDAKVIEYLSNDAKLIQSYRRLILDKYVQSNMFLCWCPGADCGRAVKSSYGDSQLITC
QCGTKFCFKCSNEWHEPVSCHHMRLWVKKCGQNSETANWILKNTKDCPKCLAQIEKNGGCNYIRCTNPAC

GFQFCWICLKAWSVHAQAWYNCNSFDQAAEKTREKFRTNLDRYIFYYNRYNGHRDSLKLESKLIRKVEQQ
MQRMQARGMSFTEVQFLRTAVDTLRICRETMMFTYVFAYYLEKNNHSLIFESNQKDLEMATETLSGYLEQ
DLQTEDLSKLKQNVQDKCAYVERRRKQLMDHCAEGDEKGHWAFSE
Caenorhabditis brenneri EGT54574.1
MSSDEEDYGEESEYETDEIEMDDDSDKFLTPSDLTAEMNLAITDAQSILQVNAGVCRLLLQKFKWNKNSL
LDKFYENPDTNEFLTKSNIIPCKLEPIGSQSDECEICCMESELVGLQCNHLACRECWTHYLSERVKADQS
EIECMTTDCKLLIPDEQIKKFICDENLKNSFDRVTINNYVEANPYLTWCPADYCSKAVKVVNTGTRLITC
PCGTIFCFTCGNDGHDPVSCRHLKLWQKKCEEVKSKATTGDGYSSDNDTFKWILSNTKDCPQCLTAIEKN
GGCNRILCRNKKCMFEFCWLCMKSWALHGYSPCNTFNEDAEKNRVDSRAELLRLQFYLNRFMEHDRSLQL
EKKLVRTVETQMEKIQDLTKSWTETQFLRKAVDVLSECRRTLKFTYAFAYYLERNNHALLFETNQKDLEM
AVEQLSGFLEKDLENDDVATLKQKVIHRVLFESDGYYKLFYFRFKIRVATWNTVARFSWIIVLKETSRTS
IIIHVL
Caenorhabditis brenneri EGT55876.1
MSSDEAENLYSDGSEAQEEIEVDDYKILDVTSIESTMKKQIGEIEILLGVSEGVGRLLLQAHKWNKDSIT
DKFYDSADKDTFLIESNIIPTDPQPFEEGEAECEICCETTELVGLDCNHRSCKECWKAYLTEKIKDGQAE
IECMDSKCKLLLEDAKVIEYLSNDEKLIQSYRRLILNKYVQSNMFLCWCPGADCGRAVKSSYGDSHQITC
PCGTKFCFKCSNEWHEPVSCHHMKLWVNKCGQNSETANWILKNTKDCPKCLAQIEKNGGCNYIRCTNPAC
GFQFCWICLKAWSVHAQAWYNCNSFDQAAEKTREKFRTNLDRYIFYYNRYVGHKDSLRLESKLIRKVEQQ
MQRMQARGMSFTEVQFLRTAVDTLRICRETMMFTYVFAYYLEKNNHSLIFESNQKDLEMATETLSGYLEQ
DLQTEDLSKLKQNVQDKCAYVERRRKQLMDHCAEGDEKGHWAFSE
Caenorhabditis brenneri EGT55883.1
MPDLLEIDEGTSKIVDTMREWNRVFPEITSDQAISVENDLMLKVRNLIITTYEVIFDFVKTKSSSLKEQK
HAGDLILTEISSRISGHEELLEKMLKTQVNQHEFNKNILDSVKSLRVHQNEQNTKICHIMTRISERDLVI
KHCGERLDELEKQNRKGGKRVRFSDVIDYKFHTNWSDLSEDLKLKCIKQMDFQDRLRLRSTAHTERRLVD
SQRFVFDEINLFHSDSFIATWNNLKCTKYQLLPRTGFLKTPEEISRIQSLTELLVYILNHCNIRNLSVEQ
LPGEHVSILEELDDLVDDNSVKIKNFRSDFFSQTTKFFLKKSWKKLDSVSIEQHGFYGKELIAIPSVAAA
KNYFLTNGQGSRRLLPLVTNFMFRNGEIGTKLVVNCMDKALFNKVISTYCWRAILHLRQTSKTEKYLVDV
EGIGMEKKKRGEQKVENLVEKDDDAAGEYCPIYGDSDDDDDKDEPDFKVRSHESLYAEMKKKIGEAQELL
EMRPGICQILLQKYKWSVAFLMEKFYDNPDRGAFLTAVNVDPSEHLHAIVGECQICFEEQELTGLSCEHR
YCWDCLREYMIDKIFDGQSEIKCIGLECPLVFEEEKIGSIVIDPEVMSCYHRLLVQKYVQNDAFMKSCPD
LSCENTIQVLNPSIRHVKCNCGYSFCFSCGNDSHEPISCRYLDKWLLKGDEDQSSVWILTNTKKCPKCNA
PIEKNGGCMHMTCHSKDCRYEFCWLCMRDWRAHANCNDFQRTNDAAREAMINKTIADRDRYKFYHDRYAG
HMQSLKLEKPLRAKFDEQMKEIEDGDRDLRDFQYIYVAIDALSASRRTLMHSYVFAFFLEENYSAIIFKS
NQADLNDATENLSKVLEEFVNSESVGFADDSKKEIMHKSQYVEQRRKALIEHCIEGDDNDDWVFNE
Caenorhabditis brenneri EGT56035.1
MDDEDMSCTSGEDYGGYGDEDYYNEADVDAADDVAVTPTHSEEADYECLSVNQVERVFIDGVNSLVSRIQ
INEKYARILLEANQWDADKIVTGYRKDRTEFMRKSHIESKPEPKRTLSATTSMLKGYCSVCAMDGYPTLP
HLSCGHCFCEHCWRGHIESRLSEGVAARIECMESECEVYAPSEFVLLLLKSLPALKVKYERFLLRDMVNS
HPQLKFCVGNDCEVIIRSTEPKPKRVTCQCCHTSFCVKCGADYHAPTSCETIRQWMTKCADDSETANYIS
AHTKDCPQCHSCIEKAGGCNHIQCTRCKHHFCWMCFGDWKSHGSEYYECSRYKENPSVAAEANHVKARRA
LEKYLHYFERFENHSKSLKMEEELRDKIRKKIDDKVNEHNGTWIDWQYLHKSVSLLTKCRYTLQYTYPFA
YYLGAGPRKNLFEYQQAQLEKEVEELAWAVERAEVTARGALEAHMHRAEHKRQVLLHDFFF
Caenorhabditis briggsae CAP38120.2
MSSTENRENEKPSKHAILDANRNEMMEKRRADSEETIEDEDVDDVEDEEEEEYDDIRFVGEESSDEENEE
EAEEDDEDGDLTAGDVAAFNDDSEKPKKNQVLTLDQLESEITGIVTDVKNILEVSPGVAQILLLKFSWNK
ELLLEKFYETSDIQQFMMDYEVIPNAMEELPQEEFGDCMICFENVLLVGLACNHLFCFGCWNSYLTEKII
DAKQSEITCMHGGCRLLLQQEQISFYITDPVVMALYNRVVVDSYVATNRLLKWCHGADCDNALKVTLKST
RHVTCNCGSSFCFSCNQDSHEPVPCRLLVLWTKNDQKDDAESFKWILGNTKECPKCQAPIEKNGGCNHMT
CNNKSCRHEFCWLCMGNWIGHQQCNVFVATGDSNREKTLANLQRFEFFKTRYLGHQQSLKLENDVNTLRT
DIRHKMRQLKEFFDLTTFQVIYLEKALNALTECRRTLMYSYIFAYYLEPNLNSKIFQLNQRDLESATEQL

SEILERKLEEDDLESLKQRVTEKYQYVEQRRQSLLDHCAEGEENDYWAYHA
Caenorhabditis briggsae CAP37079.2
MDDEDMSCTSGDDYGGYGDEDYYNEADVDAADDVEVTPTHSEEAEYECLTVSQVNRLILINHEQSPKKSD
FQVERVFLDGMNQLTARIPVNDKCARTLLEANQWDVERVVKLYRQDRTDLFRRSHIDARPEPKRKLSATS
GVKAKGYCTVCAMEGHAELPHLACGHCFCEHCWKSHVESSVRCGADYHAPTSCDTIRQWMTKCADDSETA
NYISAHTKDCPQCHSCIEKAGGCNHIQCTRCRHHFCWMCFGDWKSHGSEYYECSRYKENPSVAAEANHVK
ARRALEKYLHYFERFENHSKSLKMEEELRDKIRKKIDDKVNEHNGTWIDWQYLHKSVSLLTKCRYTLQYT
YPFAYYLTGSPRKNLFEYQQAQLEKEVEELAWAVERADGTARGALEAHMHRAEHKRQTLLHDFFF
Caenorhabditis briggsae CAP27227.2
MDSDGSDEMMYDDNREMEEEDDSDTESEEKDEKSDEFQILNPAALDSTMTTSISGVVETLEIPSGTARIL
LQKFKWNNDILMDKFYESTDVESLLKVHKIESSESQGASETGDCDICCDTGTLTGMSCGHVACYECWKMF
IMEQVKEGHSEIQCMASKCELLMPDEKVLGYLEDSEPLKSMILNNYVQTNVFLKWCPGPNCENAVKSDYC
NPHLVTCTCGTRFCFSCCDDFHNPINCRQMKLWLKKCSESGENAKWIIQNTKDCPKCLTSIEKNGGCNYM
RCTKPACGYQFCWICMDSWEVHKHAWYKCSSFDKSKDTNRKEYRSNHDRYLFFYNRFRIHVESVKLEKKL
VAKVEKLMDKMQQRSIPWAEVRFLPAAVDTLSNCRRTLTYTYVFAFYLNSNNHSIMFENNQKDLEMATEQ
LSGFLERDMEKVDDLKALNRDVQDKCRYVEHRRKVLLDHCSEGVEQGIWNFIEE
Caenorhabditis briggsae CAP27237.2
MESDSEFEMSDVGSEQGDEAEDQFLTTSELEEEMEGTISDVQSILEVSRGSCRILLRKHKWNKEHLMEHF
HDFSSLETTELLVAASNDGECPICCGVGSLSSLSCNHSACDICWEAYLSQRIVAGVGGIECMANGCKLLA
EDEKLTHTDNHPITCPCGCTFCFSCGENWHAPVSCRLLRIWNLRCIDDTESFNWINANTKDCPNCNKHIE
KAGGCNKIMCQSCRFLFCWMCLKNWDAHGYSPCNSFEVKSAKDAREQSRFDLNRYLFYYNRFLGHQRTQS
KVQVKMEQLQKRSTMTWIDVQFLPDAIEVLSACRRTMIYTYVFAFYLERNDNQSLIFESNQKDLEMATEQ
LSAILEQQLDLSGDVRMLKLKVQDTCRYVDHRRKLLLGHCTEGTDQGIWKFSE
Caenorhabditis elegans NP_491747.1
MNSDDEIYMEGSASSEDDMDDECLSDDDGIARHDQSASDYLNKKDKDNEVLDHDSLEAEMKKAISEVEAV
LQVKTGVCRILLHKYKWNKESLLERFYEHPDTIAFLIDAQVIPRQQEVIPAGDAECDICCSMDELSGLSC
NHRACAECWQAYLTNKIVSDAQSEIECMAPNCKLLIEDEKVLSYISDPTMVSKYRKLMVASYVEINCLLR
WCPGIDCGKAVKVSHWEPRLVVCSCGTCFCFSCGQNWHEPLNCRHLKKWIKKCQDDSETMNWINANTKDC
PKCMIPIEKNGGCNRMLCTNSGCRYEFCWMCLEPWTKHGYQYACNGYDETAVKNPQDAQEISRANLKRYL
FYFNRYMGHEQSLQLEGKLNIKVAKKMEQMENMSMSWIEVQFLRKAVDILSECRRTLMYTYAFAFYLKKD
NNSIIFESNQANLEMETEQLSGFLERDLEDEDLVTLKQKVQDKYRYVEQRRKVLLDHCAEGAEQDIWQYN
D
Caenorhabditis elegans NP_491748.1
MNSDAEMNTEDGGSSPEEFGEADCFSEEEDEEIVLDTSDNDTSYAKEDKKSENEVLDNDLLEAEMNTTIA
DVQAVLQVDPGVCRILLHKYKWNKESLLERLYEHPDTIAFLIDAQVIPRQQEVIPAGDAECDICCSMDEL
SGLSCNHRACAECWQAYLTNKIVSDAQSEIECMAPNCKLLIEDEKVLAYIKDPTIIAKYRKMMVASYIEI
NALLKWCPGVDCGRTVKVSHGEPRLVVCTCGSRFCFSCGQDWHEPVNCRLLKLWMKKCNDDSETSNWINS
NTKECPKCMATIEKNGGCNQITCKNTGCKFQFCWMCLGPWTVHANAWYKCNKFDDEASQTARTAQELYRA
NLTRYLFYYNRYMGHLQSLRLEGKLNKTVKAKMDQMQNLSMSWIDVQFLRKAVDVLSECRNTLMFTYIFA
FYLKRDNNSMIFESNQKDLEMETEQLSGLLERDLENEDLLTLKQKVQDTFRYVEHRRKVLLDHCAEGTEQ
DIWQYNE
Caenorhabditis elegans NP_491749.2
MSSDDEINMDDSDSSQGEIDDGCMSDDDGIVLESREQNSSDYKDNGEPDNEVLNHDSLEAEMKKTITDVQ
AVLQVKTGVCRILLHKYKWNKESLLERFYEHPDTTTFLIDAHVIPRRQERLPAGDAECDICCSLGELSGL
SCNHRACTQCWKAYLTNKIANNAQSEIECMAPNCKLLIEDEKVMFYITDPTVIATYRKLIVASYVETNRL
LKWCPGIDCGKAVRVSHWEPRLVVCSCGSRFCFSCGHDWHEPVNCRLLKLWLKKCNDDSETSNWINANTK
ECPKCMITIEKDGGCNHMTCKNTACRFEFCWMCLGPWEPHGSSWYSCNRFDDSAAKNARDAQEVSRANLQ
RYLFYYNRYMGHQQSLRLEGKLYATVKSKMEQMQTLSMSWIEVQFLRKAVDVLSECRRTLMFTYAFAFYL
KRDNNAIIFESNQKDLEMETEQLSGFLERDLDNENLVTLKQKVQDKYRYVEHRRKVLLDHCSEGADQELW
VFNE

Caenorhabditis elegans NP_500829.2
MDDEDMSCTSGDDYAGYGDEDYYNEADVDAADDVAVTPTHSEEADYECLSVNQVERVFIDGVNSLVSRIS
INEKFARILLQANHWDVDKIARLVRNDRNDFLRKCHIDAKPEPKRKLSSTQSVLAKGYCSVCAMDGYTEL
PHLTCGHCFCEHCWKSHVESRLSEGVASRIECMESECEVYAPSEFVLSIIKNSPVIKLKYERFLLRDMVN
SHPHLKFCVGNECPVIIRSTEVKPKRVTCMQCHTSFCVKCGADYHAPTSCETIKQWMTKCADDSETANYI
SAHTKDCPQCHSCIEKAGGCNHIQCTRCRHHFCWMCFGDWKSHGSEYYECSRYKENPSVAAEANHVKARR
ALEKYLHYFERFENHSKSLKMEEELRDKIRKKIDDKVNEHNGTWIDWQYLHKSVSLLTKCRYTLQYTYPF
AYFLSATPRKNLFEYQQAQLEKEVEELAWAVERADGTARGALEAHMHRAEHKRQTLLHDFFF
Caenorhabditis elegans NP_001041060.1
MSSDDEIYNENNDLDEEFSDDMDQQSGSSGESQGKANYEILDPTALESDMSKTISEVQAILQVEPGICRI
LLHKFKWNKDRLLDKFYEHSDTTEFLAEAQVIPKTSSSEEAAGSSAPPPGGDAECDVCCSMTRLSGLACA
HRACDECWKAYLTEKIVDVGQSEIECMMMDCKLLIEDEKVMSYITDPFVIAAYRKLIISSYVETNSQLKW
CPGAGCGKAVKGEPSDREPAVCTCGERFCFACAQDWHDPLSCRHMKMWRKKCSDDSETLNWINANTKPCP
KCSVTIEKNGGCNHMSCKSSSCRYEFCWLCLGDWKNHAQCNRYVEDDNKTDSRSLSRKNLQRYLFYYNRF
MAHQNSMKLEGKLYAKVEVKMDLMQALSMSWIEVQFLRRAVDALCECRRTLKYAYAFAYYLEANNMTTLF
ETNQSDLELATEQLSGMLEGDLEDNDLAELKRKVQDKYRYVELRRKKMLDHCAEGVELDSWVFCE
Caenorhabditis japonica CJA07979
MKTQCLSDDDGIALESHEQNSLDFKDGSTANEVLNHDQLETEMKKTIGDVQAVLQVKSGVCRILLHKYKW
NKESLLERFYEHPDTNAFLIDAQVIPRRTEKLPRGDAECDICCMLSDLSGLACNHRACTPCWKAYLTNKI
VDGGQSEIECMAANCKLLIEDEKVMSYITDPIVIASYRRLIVASYVETNRLLKWCPGVDCGKAVKVNHCE
PRLVVCTCGSRFCFSCGNDWHEPVNCRLLKLWMKKCSDDSETSNWINANTKECPKCLITIEKDGGCNHMT
CKNTACRFEFCWMCLGPWEPHGSSWYSCNRFDDSAAKSARDAQEVSRANLQRYLFYYNRYIGHQQSLRLE
GKLYATVKSKMEQMQQLSMSWIEVQFLRKAVDVLSECRRTLMFTYAFAFYLKRDNNAIIFETNQKDLEME
TEQLSGFLERDLENENLVTLKQKVQDKYRYVEHRRKILLDHCSEGAEQDIWVFNE
Caenorhabditis japonica CJA10833
MESECEVYAPSEFVFVLLESFPFLKSKYERFLLRDMVNAHPQLKFCVGNDCSVIVRSTEAKPKRVTCQQC
HTSFCVKCGADYHAPTSCQTIKEWMTKCADDSETANYISAHTKDCPQCHSCIEKAGGCNHIQCTRCRHHF
CWMCFGDWKSHGSEYYECSRYKENPSVAAEANHVKARRALEKYLHYFERFENHSKSLKMEEELRDKIRKK
IDDKVNEHNGTWIDWQYLHKSVSLLTKCRYTLQYTYPFAYYLQAGPRKNL
Caenorhabditis japonica CJA39226
MSYITDPIVIASYRRLIVASYVETNRLLKWCPGVDCGKAVKVNHCEPRLVVCTCGSRFCFSCGNDWHEPV
NCRLLKLWMKKCSDDSENVEIGLKCLSDDDGIALESHEQNSLDFKDGSTANEVLNHDQLETEMKKTIGDV
QAVLQVKSGVCRILLHKYKWNKESLLERFYEHPDTNAFLIDAQVIPRRTEKLPRGDAECDICCMLSDLSG
LACNHRAX
Caenorhabditis latens OZF95120.1
MSSDDDMYDDDSDFEQETVDDQILDQEGLKSDMEEAITSVQGTIQVTVGVARILLQTHKWNQDALIDKFY
DSADLETFLSAANIPLQSVPLVDGECDICCDEAPLTGLSCAHLACSQCWKAYLTEKIKEGQSEIECMAPK
CQLIIPDEQVVKCISDDTKYHQVILNNYVKANVYLEWCPGIDCGKAVKASNCDPHLIVCTCGTRFCFACS
NDWHEPVDCRQMKLWVKKCGESSETATWIIENTKDCPKCLTSIQKNGGCNYIRCTNPKCGYQFCWICMNA
WSNNRSQFRNNHDRYLFFYNRYRTHEQSLKMEERLIAKMNLKMEQMQNHDMTWTEVQFLREAVNVLSLAR
RTMMFTYVFAYFLQKNNHSMIFETNQKDMEMATEQLSGFLEQDLEKENLQTLKQKVQDKCRYVEQRRNAL
LNHCKEGVEQGVWEFIEWF
Caenorhabditis latens OZF96919.1
MDDEDMSCTSGDDYGGYGDEDYYNEADVDAADDVAVTPTHSEEADYECLNVNQVERVFTDGVNSLVSRVP
VNEKFARILLEANQWDVEKIVKHFRQDRTEFMRRSHIDARPEQKRKLSATATSIAKGYCSVCAMDGYSEL
PHLSCGHCFCEHCWKSHIESRLSEGVAARIECMESNCEVYAPAEFVLKIIKSSNALKLKYERFLLRDMVN
SHPHLKFCVGNDCQVIIRSTEIKPKRVTCLSCHTSVKCGADYHAPTSCDTIRQWMTKCADDSETANYISA
HTKDCPQCHSCIEKAGGCNHIQCTRCRHHFCWMCFGDWKSHGSEYYECSRYKENPSVAAEANHVKARRAL
EKYLHYFERFENHSKSLKMEEELRDKIRKKIDDKVNEHNGTWIDWQYLHKSVSLLTKCRYTLQYTYPFAY
YLGAGPRKNLFEYQQAQLEKEVEELAWAVERADGTARGALEAHMHRAEHKRQTLLHDFFF

Caenorhabditis latens   OZG08984.1
MDSDEDVYMVESDSDDGYPEDEILSFEDLESEMKASISEIQDVIEGSTDICRLLLQKYKWNKDFMLDRFY
ESPDTLAFLIDANIVPKQSAVLSEGDAECQICCMEGNLSGLACNHLACDYCWKAYLTEKIKEKQSEIECM
TSNCKLLMKDEQVKKYLGDSASIASFRRILVNSYVKVNSSLRWCPGENCEKAVKVHQPSESRLLICSCGT
RFCFTCGNEGHEPIDCSYLKLWLKRCMDDSETFNWINANTKDCPKCSAPIEKNGGCNYMRCENTKCRYEF
CWMCFGSWKNEGAHSCNTFKEKETKNPTRDKSRLKERVSLKMTEMQEKSMTWVEVQFLPKAVEVLSECRH
TLMFTYAFAFYLKKNNSSIIFEENQKDLEQSTEQLSGFLERDLDNEDLVTLKVKVQDKYRYVEQRRKALL
DHCAEGKEQNVWVFNE
Caenorhabditis latens   OZG09200.1
MSYSDDEIQIDDSDTDQGDMGDECISDDDGIALESHDQNNSEYRENAAPDNEVLNHDSLEAEMKKAIADV
QAVLQVKTGVCRILLHKYKWNKESLLERFYEHPDTTTFLIDAQVIPRLTQTVPTGESECDICCMVSGLSG
LACNHRACTPCWRSYLTNKIVDGGQSEIECMAANCKLLIEDEKVMLYIKDPDVIASYRRLIVASYVETNR
LLKWCPGVDCGKAVRVGHCEPRLVVCSCGSRFCFSCGNDWHEPVNCRLLKLWMKKCSDDSETSNWINANT
KECPKCMITIEKDGGCNHMTCKNTACRFEFCWMCLGPWEPHGSSWYSCNRFDDSVAKTARDAQEVSRANL
QRYLFYYNRYMGHQQSLRLEGKLYATVKSKMEQMQTLSMSWIEVQFLRKAVDVLSECRRTLMFTYAFAFY
LKRDNNAIIFETNQKDLEMETEQLSGFLERDLDNENLVTLKQKVQDKYRYVEHRRKILLDHCAEGAEQDI
WVFNE
Caenorhabditis latens   OZG09572.1
MSSDEMDLYDSDIGEEAEGVEKIEFLNREDVELEMKKLISEVESIVEVNAGMCRNLLHKFKWNKDALLNK
MYESVDIQQFLIDSQVMAKCDDKVEEAREGECDICCSIGVLTGLDCNHLACNDCWNMYLKEKIVDNGICE
IECMEPDCNLLMEESKIGNYTTNPFILAKYRFQSINGYVDASSRLKWCPGIDCGRIVKIPDGQIRLVVCK
CETRFCFNCCEDFHDPIDCRLMKKWLKKCSDDSETSNWMNANTKDCPKCLVPIEKNGGCNHMRCTNIKCK
LEFCWMCMKPYKDHQVSGYTCNRFDESKEKNRSETRALLERWLFYHNRYMNHLQSLKLEEKLKEKVSAKE
EELQKNSGMTWVEVQFLSQSVSALSECRRTLMYTYAFAFYLKKNNNSEIFESNQRDLEMATENISGYLER
ELETKDLGTVRQKVQDLSRYVGQRRKALLDHCEEGVENGFWDFLDSK
Caenorhabditis latens   OZG11131.1
MNSDDEIEFDASDSEPEVEEVKYQSLTRDALIAEMNEAIEEVESVIQVPPGTCRILLHKYKWNKDSLLER
FYEKSDTKEFLIDSQVIPKVTVDSKNEEAECVICCDLVELTGLSCNHRACNNCWTMYIMDKIKDGQSEIE
CMASDCKLLMEDEKILEYITDKEAITKYRDLVVDSYEYHDPVGCRHLKMWNKKAQEMKDRKHNGEGYGAD
KETFTWLMSNTRDCPKCLVSIEKNGGCNYMLCKNPKCRFQFCWICMNAWSVHSNAWYKCNSYDEEADKKR
EASRADLHRFLFYYTRYFNHKRSLDLEQKLRIIVRTKMEELERKQMRWIEVQFLETAVGVLSECRKTLLL
TYIFAYYLKKDNNTAIFEGNQKDLELATEQLSGFLERDLEQEDLTALRLKVQDKCRYVEHRRKILLDHCS
EGYEQDFWEFSE
Caenorhabditis latens   OZG13999.1
MNTENLENEGLPDESKFGDNWQGVQKIKADDRNCMGDATNVDECRDEAKETDDEIEYEESEGLIDEEDED
DECIESCDNDTVYFKNNKILSLDKLETEMKEIISDVETILEVSTGISQNLLQKFRWNKETLLEKFYGSDD
TNEFLMNQNVIPSDPEDFPSEENTQCDICADDESVLTGLSCNHQFCIGCWNSYLTQKIVEGGETEISCMA
PECPLLFQQEQITFYINDPTVMSMYRKAVVSNYVDTNRLLKWCHGAGCEKVIKVPHASIRHVACSCGSQF
CFSCNKDSHEPASCHILTHWLKMDDQESSKWILSNTKDCPKCQAPIEKNGGCNHMTCTNRNCRYEFCWLC
MGDWRNHQNCNQYQPEPGSKREKHLANLERYAFYNGRYLAHQQSLNLEENLREEIKSKMSKLQEFFALSK
PEVLFLQKALNALSQCRRTLMYSYVFAFYLEPNFNSIIFEANQQDLQSATEQLSEILERKLEDDDLDSLK
QRIQDKYQYVETRRKCLLDHCAEGEEKDDWVYIEY
Caenorhabditis latens   OZG15559.1
MDSDDEIQFNDSGSDGESEQKGTQILSFADLETKMKDAISEIQDILEVKPGVCRILLQKHKWDKDSLLER
FYEHPDTNEFLKAANVIPEESETFPELPVPTDCDICCMPGELTGLACGHLACIDCWKAYISDRINDGKCE
VECMTGECKLLMEDEKVLFYITDPIILEKRRQLIVNSYVEISKCLRWCPGKNCGKVIKAAHSEPHLVQCS
CGTQFCFFCGNDGHEPVSCRLMKLWEKKCLDDSETANWISVHTKDCPKCLAPIEKISGCNRMLCRNPSCK
FQFCWMCMRDWDVHGYSPCNSRAYINALLSYDPKKEKDRVLQHHSQISWADVQYLPKAVEILSTCRRTMM
NTYIFAFYLEHNNHAEMFEANQKDLEMATEQLSGFLEQDLLSQSGQEKMKTLIQNVQDKCRYVEHRRKIL
MDHVVEGTEQDVWVFREE

Caenorhabditis latens OZG17676.1
MRYGANALRISRTLPTVPAVSAAKAEIGICGSGMPHAMSSDDEFSLEDSENDASDFESETENLETEDQDE
EVVRVLTYSDIREIMKSKIEEVREILEVTSGVCRVLLQKYKWDKTTLLEKFYEDPNFIVNSKISLTMRTD
SSDSSSDGECDICCDTAPLLGLSCDHTACIECWRAYLTEKINEKKCEIQCMSSDCKLIIDDDKIHEYLSS
ETTVISAFQQLTVDEYVETNHFLTQCSCGMVLESSRSDAHLVVCSCGTRFCFSCGNDSHEPVNCRLLKLW
EKKCVGVKDKAGADGYSSDKETFNWILSNTKDCPKCVTSIEKNGGCNRITCRSKTCRFEFCWLFNEKDEK
NRVDSRAELHRFLFFYNRFKSHEQSFKLEKKLVKTVNVKMDEMQKKGMGWADVLFLRKSVDILSECRRTL
MFTYIFAFYLERNNQAIMFDGNQKDLEMAVEQLSGLLEQEMETNDLRVLIQKTQDKSRYVEYRRKVLLDH
CTEGMEQDAWVYNA
Caenorhabditis latens OZG20306.1
MDDRYNLENRNEVIEQVIDMFNYVTKIILNLNSIFHCFICFFLSSQYREVVWKLLRLDKRKKILTFSDIQ
KAMQSQITDIQSVFKMSNGECRVWLQKYNWDKEKVMEHFYENPMNSKNSNESSVSPENGICDICCEETQL
IGLHCNHLACLECWRAYLAENIKEGKSEIGCIGSNCEAIIYDEKIREFLEDPKILEGFVRNTVNAYVETA
KCLVWCPGTNCGNAIKSLDPHHVTCSCGTRFCFSCGQNPHEPVTCALLNTWSKKCLTEKNNISGAEYSSD
METLIWVLSNTKDCPKCNTAIEKNGGCNKMTCRSATCRFKFCWLCLKDWTEHGYSGYCNVFTDTDETRDS
RADLLRFLFFYNRFKAHEQSLELEKKLRAKVSSQLDGTQGLSWSQVKSLEKTIDILSESRHTLMHTYIFA
YYLEQNNQSIIFESNQKDLEMATEQLSGYLEQDWEAYFHKILDKSRYVEHRRNVLLEHCAQGDGLEMNTT
GVSKRKKSSGFKRRRPWRHGVLLLAIVVIFIINFFLWGHIFYQSTSTVCVILPE
Caenorhabditis nigoni PIC33358.1
MDDEDMSCTSGDDYGGYGDEDYYNEADVDAADDVEVTPTHSEEAEYECLTVSQVERVFLDGVNQLIARIP
VNDKCARTLLEANQWDVERVVKLYRQDRTDLFRRSHIDARPEPKRKLSATSGVKAKGYCTVCAMEGHAEL
PHLACGHCFCEHCWKSHVESRLSEGVASRIECMESNCEVYAPQEFVLVIIKSSPALKQKYDRFLLRDMVN
SHPHLKFCVGNDCQVIIRSTEAKPKRVTCLACHTSSCVRCGADYHAPTSCDTIRQWMTKCADDSETANYI
SAHTKDCPQCHSCIEKAGGCNHIQCTRCRHHFCWMCFGDWKSHGSEYYECSRYKENPSVAAEANHVKARR
ALEKYLHYFERFENHSKSLKMEEELRDKIRKKIDDKVNEHNGTWIDWQYLHKSVSLLTKCRYTLQYTYPF
AYYLTGSPRKNLVSFLKTNLAKMRKYKTPKTHNWKTGHIFFRFDIFGLTKSDKIEIEEYYKIILFGILYL
VYEQSYKPKSCDRLSHTGVGL
Caenorhabditis nigoni PIC36175.1
MVPQRLLQRRADSEETIEDEDIEEEGVEDEEEEYDDIRFVGEESEDEEKEEAEESEDDDDYKASGDVAAF
DVDSEKPKGNQVLTLDQLESEVTGIVTDVKNILEVSPGVAQILLLKFSWNKELLLEKFYETSDIQQFMID
NEVIPSVMEELPQEEFGDCMICFENVLLVGLACNHLFCFGCWNSYLTEKIIDAKQSEITCMHGGCKLLFQ
QEQISFYITDPVVMALYNRAVVDSYVATNRLLKWCHGADCDNALKVTLKSTRHVACTCGSSFCFSCNQDS
HEPVPCRLLVLWTKNDQKDDAESFKWILGNTKECPKCQAPIEKNGGCNHMTCTNKSCRYEFCWLCMGNWI
GHRQCNVFVATGDSNREKTLANLQRFEFFKTRYLGHQQSLKYENDLRTEIIFKMEQLKEFFDLASSEVVY
LEKALKVLTECRRTLMYSYIFAYYLEPNFNSEIFEGNQQDLQSATEQLSEILERKLEDDDLDSLKQRVME
KYQYVEQRRQCLLDHCAEGEENDYWAYHA
Caenorhabditis nigoni PIC42084.1
METDEELFMEDSDSEPEFLEDELQVMKFEDLEEEMKVAIADLQDVLEVSPDICRVLLQKFKWNKDALLDR
FYESSDAVSFLIDAHVLPSRSVSESEEEECQICCMEGRLTGPACNHKACTECWKAYVTEKIKEGQSEIEC
MTSNCKLIIEDSQVEQFIGDPIGIASYRRVLVNSFVRVSKNIKWCPGENCLKAVKVHQPSDSRLIVCPCG
TRFCFTCGNEGHEPIDCNYLKKWLKRCMDDSETFNWIHANTKDCPKCSAPIEKNGGCNYMRCENTACRYE
FCWMCFGAWKNEGAHSCNTFREKNAGGKTDREKSRVSLERYLFYYNRYIGHERSLKLEKKLKEKIARKME
EMQQLSMTWVEVQFLQKAVEVLSECRHTLKYTYAFAFYLKRENNAMMFEANQNDLEQATEQLSGFLERDL
DRENLITLRQKVQDKSRYVEQRRKALLDHCNEGNDENIWVFNE
Caenorhabditis nigoni PIC48835.1
MDSDYSGDEEYVEENDEDLSDEDQVVAMKVEDNKFMTHGELEIEMKKTIAEVQAVLQIKKGICRILLHKF
KWNKEALFDKFYDCLDMTEFLKGCQIIRKSEDEESHKGECDICVDLTDLVGLSCGHLACSKCWKAYITEK
IVDGRQSEIECLASKCDLLIEDEKIKTILSDSLVLDAYDYHKVNGFVMTNPFIKWCPGKNCGRAVKVFST
DSQLIECECGARFCFCCYENWHEPVTCELQKQWVIRCKSDSETANWIMAHTKECPKCHTTIEKNGGCNHM
SCQTRSCMHEFCWLCMKPWRGHTACNSYVDKNEQQRTNSRQSLERYLFYYNRYRTHEQSLQLEMKLKKKV

AIKMDQIANATKAYVEGKCLEQAVEVLSQCRQTMMYSYVFAFYLQKDNNSQIFEDNQKDLEAATENLSEL
LEKDIDEDDYSKLFKLVQDKSRYVDARRKVLLEHCIEGYDKNFWKFTTAAFR
Caenorhabditis nigoni PIC49584.1
MDSDGSDEMMYDDNRDMQEEDSSGTESEEKYEKSDEILDPTALECTMTASISGVVETLGIPSGTARVLLQ
KFKWNNDALMDKFYESTDAENLLKAYKIESSESQGSSETGDCDICCDTSTLTGMSCGHVACNECWKTFIM
EQVKEGHSEIQCMASKCELLMPDEKVMGYLEDSEPLKRMIINNYVQTNVFLKWCPGPNCENAVKSDYCNP
HLVTCTCGTRYCFSCCDDFHSPINCRQMKLWVKKCSESGENATWIIQNTKDCPKCLTSIEKNGGCNYMRC
TKPACGYQFCWICMNDWEVHKHAWYNCSSFDKAKETDRKEYRSNHDRYLFFYNRYRIHVESVKLEKKLVK
KVEKLMDKMQELSIPWSEVLYLRAAVDTLSNCRRTLMYTYVFAFYLNSNNHSIMFENNQKDLEMATEQLS
GFLERDMEKVDDLKALNRDVQDKCRYVEHRRKVLLDHCSEGVEQGIWDFIEE
Caenorhabditis nigoni PIC49586.1
MDSDGSEEMMYDANDNWDVQDEESGKKSEKNEENSDESVMDSQDEWDNDDDDVEQVDSDDDDSEEEKKWI
IKEPVVENTILTYSEILASMQAIIKDVQSVLQQPAGICRILLQNHKWSKGQLLERFYEDPEFLTNTNMIP
KDPAQKPEDGPRDCDICCENTELVGLSCNHMACKDCWKFYLAEKIKEGKSTIECMASNCKLLVYDFNEFV
GDDKEMIAQFEKLIVNAYVESTSSISWCPAENCSLAVKSDSNGIVECSCGTKFCSSCGSAPHDPATCRHV
KIWNRKAEKEKATSSVGFSTDKDTFKWILSNTKDCPKCMTAIEKNGGCMRMSCRNKSCRFEFCWMCLRQW
SVHGYSPCNTFSEEAEKNRLDSRAELLRFMFFYNRFKAHEQSLGLEKKLIQKIEKKMDEMQTGGLCYSET
LFLRKAVDTLSTCRRTLQYTYVFAYFLERNNHAIIFENNQNDLEMATEQLSGFLEQKLDAVTDLKVLSRN
VQDKARYVEHRRKVLSEHCVEGLEMENWVFTG
Caenorhabditis nigoni PIC52870.1
MSSEDEIQMDDSDSDQGDLDDECLSDDDGIALESHDQNNSEYRENAVPDNEVLNHDSLEIEMKKTIADVQ
AVLQTKSGMCRILLHKYKWNKESLLERFYENPDTTTFLIDAQVIPRHTESVPSGDSECDICCIVGPLSGL
ACNHRACTACWKSYLTNKIVDAGQSEIECMAANCKLLIEDEKVMTYITDPNVIASYRRLIVASYVETNRL
LKWCPGVDCGKAVKVSHCEPRLVVCSCGSRFCFSCGNDWHEPVNCRLLKLWMKKCSDDSETSNWINANTK
ECPKCMITIEKDGGCNHMTCKNTACRFEFCWMCLGPWEPHGSSWYNCNRFDDSVAKTARDAQEVSRANLQ
RYLFYYNRYMGHQQSLRLEGKLYATVKSKMEQMQTLSMSWIEVQFLRKAVDVLSECRRTLMFTYAFAFYL
QRDNNSIIFETNQKDLEMETEQLSGFLERDLDSENLVTLKQKVQDKYRYVEHRRKILLDHCAEGAEQDIW
MFNE
Caenorhabditis nigoni PIC53269.1
MASDSDEYSIPFEDDIDYDEVEEMEEEMEDLTISDDDDFEEVDEEGNVNPINKNLYEEELEFETNSEPEP
EEIKDEILTLNDVPSVIVKYISDIREIIEVPDGIIRILLQKFKWDKDPLLERFYEADDLYDFLRKQKIDP
FTSSQWSGTEGECEICCETFLLTGPSCNHKACPTCWKFYISERIKEGNSEIECMASKCELLLPDEQVLEY
FNDKSELDAYLGQVINSFVQFNAQMRWCPGVDCGRIVKSSSTDPHDVSCQCGTQFCFSCGNDGHSPVNCR
HLKLWLKKCLDDSETANWINANTKDCPKCLVPIEKNGGCNYIRCRNPTCRHTFCWICMKDWAIHTLGHFR
CNIFVSPEETAAAELARGQSRSFLQRYLFYFSRYQTHKHSLSLEKKLIEKMNKKIEEAQEYAMSWNDAQF
LRDAVDSLSKSRRTMMYTYVFAFYLKRNNHAEIFETNQRDLEMATEQLSGYLEQDMDEEDPKTLKQKVQD
KCRYIEHRRKVLMNHCSEGNEQEIWEFTQ
Caenorhabditis nigoni PIC53308.1
MASDSDEYSLPFEDDIDYDEVVEMEEEMEDLTISDDDDFEEVDEEGNVNPRNKNLYEEELEFETNSDNEP
EEIKDEILTLEDAPSVVVKYISDIREIIEVPDGIIRILLQKFKWNKDPLLERFYEAEDAHDFLRKQKIDP
ATSIQWSGTEGECEICCETFLLTGPSCSHKACPTCWKFYISERIKEGNSEIECMASKCEVLLPDEQVMEY
FNEGTELVAYQRQVINNFVQFNAQMRWCPGVDCGRMVKSSSKDPHVVSCQCGTQFCFSCGNDGHSPVHCR
HMKLWLKKCLDDSETSNWMNANTKDCPKCSAPIEKNGGCNYIMCTNRTCRYQFCWICMNNWMVHANGWYS
CNAFVPTLESALAESARDHAKAILERYLFYFNRYQGHLHSLSLEKKLIEKMEKKIEDLQQNSMTWNEAQF
LRHSVDILSKSRRTMMYTYVFAFYLKRNNHAEIFETNQRDLEMATEQLSKYLEQDMDQEDPKTLKQKIQD
KCRYIEHRRQVLLKHCSEGNEQEIWEFTE
Caenorhabditis remanei OZF78256.1
MSSDELELYDSDIGEEIEEVEKIEFLNREDVELEMKTLISDVESIVEVNAGMCRNLLHKFKWNKEALLNK
MYESGDTQQFLIDSQVMAKCDDKVKEAKEGDCDICCSSGVLIGLDCNHMACKECWKMYLKEKIVDNGICE
IECMEPDCNLLMEESKIANYTTNSFILAKYRYQSINGYVAASSRLKWCPGNDCGRIVKIPDAETRLIMCK

CETRFCFNCCLEFHDPIDCRLMKKWLKKCSDDSETSNWMNTNTKDCPKCSVPIEKNGGCNHMRCTNNKCK
HEFCWMCMKAWQYHKENGYKCNRFDESKEKSRSETRALLERWLFYYNRYMNHLQSLQLEEKLKVKVSAKE
EELQKNSTMTWVDVQFLSKSVSALSECRRTLMYTYAFAFYLKKNNNSEIFESNQRDLEMATENISGYLER
ELETKDLKTLRQKVQDLSRYVDQRRKALLDHCEEGVENDFWDFSE
Caenorhabditis remanei OZF88273.1
MSSDDDMYDDDSDFEQETVDDQILDREGLKSDMEEVIASVQETIQVTKGVCRILLQNHKWNQEALVDKFY
DSADLETFLSAANIPLHTPSSADGECDICCDMAPLTGLSCAHLACSQCWKAYLTEKIKEGQSEIECMAPK
CQLIIPDEQVVTCISDDTKVLETYHRVILNNYVKTNVYLEWCPGIDCGKAVKGSTCDPHLIVCTCGTRFC
FACSNDWHEPVDCRQMKLWVKKCGESSETATWIIENTKDCPKCLTSIQKNGGCNYIRCTNPKCGYQFCWI
CMNAWSSLKMEERLIAKMNMKMEQMQNHSMTWTEVQFLREAVNVLSLARRTMMFTYVFAYFLRKNNHSMM
FETNQKDMEMATEQLSGFLEQDLEGENLKTLKLKVQDKCRYVEQRRNALLNHCKEGIEQDVWEFIE
Caenorhabditis remanei OZF90757.1
MDSDDDIQLNDSDSDGESEEKGTQILSFADLETKMKDAISEIQDILEVKPGVCRILLQKHKWDKNSLLER
FYEHPDTNEFLKAANVIPEDSETFPELPVPTDCDICCMPGELTGLACGHLACIDCWRAYISDRINDGKCE
VECMTGECMLLMEDEKVNFYITDPIILEKRRQLIVNSYVEINKCLRWCPGKNCGKIIKAAHSEPHLVQCS
CGTQFCFFCGNDGHEPVSCRLLKLWEKKCLDDSETANWISVHTKDCPKCLAPIEKISGCNRMLCRNPSCK
FQFCWMCMRDWDVHGYSPCNSYDPKKEKDRVKNRANLDRYLFYYNRYKGHGDTVETKMEVLQHHSQISWA
DVQYLPKAVETLSTCRRTMMNTYIFAFYLEHNNHAEMFEANQRDLEMATEQLSGFLEQDLLTQSGQEKMK
TLIQNVQDKCRYVEHRRKILMDHVVEGTEQDVWVFREE
Caenorhabditis remanei OZF91249.1
MNSDDEIEFDASESEPEIDEVKYQSLTRDALAAEMNEAIEEVESVIQVPPGTCRILLHKYKWNKDSLLER
FYEKSDTNEFLIDSQVIPKVKKTFDSKNEEAECEICCDLVELTGLACNHRACNNCWTMYIMDKIKDGQSE
IECMASDCKLLMEDEKILEYITDKEAITKYRDLVVDSYVEINNLLCWCPNAKCGKAIRVKINEPQLVVCD
CGTQCCFSCTEEYHDPVGCRHLKMWNKKAQEMKDRKHNGEGYGADKETFTWLMSNTRDCPKCLVSIEKNG
GCNYMLCKNPKCRFQFCWICMNAWSVHSNAWYKCNSYDEEADKKREASRADLHRFLFYYTRYFNHKRSLD
LEQKLRIIVRTKMEELERKQMRWIEVQFLETAVGVLSKCRKTLLLTYIFAYYLKKDNNTAIFEGNQKDLE
MATEQLSGFLERDLEQEDLTALRLKVQDKCRYVEHRRKILLDHCSEGYEQDFWEFSE
Caenorhabditis remanei OZF91252.1
MNSDYESSDNEEQEDESSVQFLPPSTLESEMKEMIADVQSVLEIKTGVCRILLHKYKWNKDSLFDKFYEH
PDTTAFLIDAQVIPKPSPTPFPIVPNIPQECEICCELTEKLSGLACNHKACFDCWKSYLTEKIVEGRQCE
IECMDSNCQLLIEDEKVMCYITDSTVVAMYEKLTINSYVAANQYLKWCPGVDCGLAVKTTSTEPTFITCP
CGANFCFSCCQDWHEPINCHLLKKWQKRCSDDAETCNWILAHTKECPKCQVIIEKNGGCNHMTCRNRSCN
YQFCWLCMGSWSGHATAGCNSFEDEKTALRQKSRVSLDRYLFYYHRHEGHRQSLLLEKNLQEKIAVKMED
LQKIGRITWVEVKFLEQAVQVLSTCRRTLMNTYAFAFYLKRDNHAVIFEANQRDLEMATETLSGFLEQEF
DETGAY
Caenorhabditis remanei OZF97647.1
MDDEDMSCTSGDDYGGYGDEDYYNEADVDAADDVAVTPTHSEEADYECLNVNQVERVFTDGVNSLVSRVP
VNEKFARILLEANQWDVEKIVKHFRQDRTEFMRRSHIDARPEPRRKLSATVTSIAKGYCSVCAMDGYSEL
PHLSCGHCFCEHCWKSHIESRLSEGVAARIECMESNCEVYAPAEFVLKIIKSSNALKLKYERFLLRDMVN
SHPHLKFCVGNDCQVIIRSTEIKPKRVTCLSCHTSFCVKCGADYHAPTSCDTIRQWMTKCADDSETANYI
SAHTKDCPQCHSCIEKAGGCNHIQCTRCRHHFCWMCFGDWKSHGSEYYECSRYKENPSVAAEANHVKARR
ALEKYLHYFERFENHSKSLKMEEELRDKIRKKIDDKVNEHNGTWIDWQYLHKSVSLLTKCRYTLQYTYPF
AYYLSAGPRKNLFEYQQAQLEKEVEELAWAVERADGTARGALEAHMHRAEHKRQTLLHDFFF
Caenorhabditis remanei OZG03419.1
MRYLFLTTPKPGTSTERWLGKESGDYYYFNGGPVENWDTRPDYIFSEDTKQKTIASTELIDHIVSFXGLF
LNLIHFFILTRKALRQNVVFVIIIGICVCDMQIFLTSITERLCGYRARRAPFEGFCETPKEYRYYYCEMH
SKAFQTFGRLASAVLALSAAIIRAVSVLFPMSSFVDNLVKVRTGKRIFLVTWFACGVRYWQAYTDFGFWT
GSSYDGRCMIAPEFGSKTSYIYQEGYIVLVLTVLYLIVTVVLLAALAMTQRRRKKLGNDKGSSTSMLVVM
MAVSFLISESIYSIRQIIDMFNYVIKIILNLNSIFHCFICFFLSSQYREVVWKLLRLDKRKQVSLVGKKD
IYGTSDSLSISAGEGMDSNDEWSDFDIDSDDHNDDDGKKNEKSILTFSDIHESMQSQIADIQSVFGMSNG

ECRFWLQKFNWDKEKVMEHFYENPDSKNSNESSVSPAEDQICDICCEETQLIGLHCNHLACLECWKAYLA
ENIKEGKSEIGCIGSNCELIIHDEKIQEFLDDPKILEGFVRNTVNAYVETSRCLTWCPGTNCGNAIKSLN
QDPHHVTCSCGTRFCFSCGQNPHEPVTCALLKIWRKKCLKEQDNISGAEYSSDKETLHWVLSNTKDCPKC
NTAIEKNGGCNKMTCRSAKCRYKFCWLCLKDWAVHGYGHCNVFTQDTEETRDSRAHLLRFLFFYNRFKAH
EQSLELEKKLRSKVSSQLEDSEGISCSDVKSLEKTIDILSESRHTLMYTYIFAYYLEQNNQSIIFESNQK
DLEMATEQLSGYLEQDWNFTNKSDFHKILDKSRYVEHRRNVLLEHCAEGLELNTTGVSKRKKSSGFKRRR
SWRTAVVVLAIM
Caenorhabditis sinica   Csp5_scaffold_00051.g2623.t1
MSTDEEVVYMEDSDTEPDYLEDEVLRFEDLAEDMKSTIVDLQAVLEVSADICRILLQMYKWNKDALLDRF
YGSGDPVAFLIAAHVIPGRSAPENSESECQICCMDDCELSGLGCNHKACADCWKSYVTEKVKEGVSEIQC
MTSDCKLIMEDSLVEKYIGDPIGIASFRRALVNSYVSVSKTIRWCPGKNCAKAVRVHQPSESRLIVCPCG
TRFCFSCGNEGHEPINCHLLKIWLKRCMDDSETFNWINANTKDCPKCAAPIEKNGGCNYMRCENNRCRYE
FCWLCFGSWKNEGAHSCNAYKNGGTTSEKDKSRISLERYLFYYNRYAGHQKSLKLEEKLKAKVVAKMDEM
QQKSMSWVEVQFLQKAVEVLSDCRHTLMYTYAFAFYLKRDNNAMIFEANQNDLEQSTEQLSGFLERDLEN
EDLTTLKQKVQDKFRYVEQRRHVLLDHCNEGNDQGFWVFNE
Caenorhabditis sinica   Csp5_scaffold_00375.g10383.t1
MSSDDEIQIDDSESDQGELDEECLSDDDGIALESHDQNGSEYRDNAVPDNEVLNHDSLEAEMKKTIADVQ
AVLQTKTGMCRILLHKYKWNKESLLERFYEHPDTTAFLIDAQVIPRRTETLPSTDSECDICCTVGALSGL
ACNHRACTACWRSYLTNKIVDAGQSEIECMAANCKLLIEDEKVMFYINDPIVIASYRRLIVASYVETNRL
LKWCPGVDCGKAVRVSHCEPRLVVCSCGSRFCFSCGNDWHEPVNCRLLKLWMKKCSDDSETSNWINANTK
ECPKCMITIEKDGGCNHMTCKNTTCRFEFCWMCLGPWEPHGSSWYNCNRFDDTVAKTARDAQEVSRANLQ
RYLFYYNRYMGHQQSLRLEGKLYATVKSKMEQMQTLSMSWIEVQFLRKAVDVLSECRRTLMFTYAFAFYL
KRDNNAIIFETNQKDLEMETEQLSGFLERDLDNENLVTLKQKVQDKYRYVEHRRKILLDHCAEGAEQDLW
SFNE
Caenorhabditis sinica   Csp5_scaffold_00418.g11086.t2
MDDEDMSCTSGDDYGGYGDEDYYNEADVDAVDDVAVTPTHSEDAEYECLSVGQVERVFIDGVTALVARIP
VNEKFARQLLEANHWDVEKIVKLFRQDRAELFRRSHIDARPEPKRKLSATASTIAKGYCTVCAMDGYTEL
PHLACGHCFCELCWKGHIESRLSEGVASRIECMESNCEVYAPAEFVMIIIKSSPILKQKYERFLLRDMVQ
SHPHLKFCVGNDCQVIIRSTELKGKRVTCLACHTSFCVKCGADYHAPTSCDTIRQWMTKCADDSETANYI
SAHTKDCPQCHSCIEKAGGCNHIQCTRCRHHFCWMCFGDWKSHGSEYYECSRYKENPSVAAEANHVKARR
ALEKYLHYFERFENHSKSLKMEEELRDKIRKKIDDKVNEHNGTWIDWQYLHKSVSLLTKCRYTLQYTYPF
AYYLTASPRKNLNRTILPRIWLPEFSNFENRNSKLQKSSIPSLIYDFEYQQAQLEKEVEELAWAVERADG
TARGALEAHMHRAEHKRQTLLHDFFF
Caenorhabditis sinica   Csp5_scaffold_00418.g11086.t1
MDDEDMSCTSGDDYGGYGDEDYYNEADVDAVDDVAVTPTHSEDAEYECLSVGQVERVFIDGVTALVARIP
VNEKFARQLLEANHWDVEKIVKLFRQDRAELFRRSHIDARPEPKRKLSATASTIAKGYCTVCAMDGYTEL
PHLACGHCFCELCWKGHIESRLSEGVASRIECMESNCEVYAPAEFVMIIIKSSPILKQKYERFLLRDMVQ
SHPHLKFCVGNDCQVIIRSTELKGKRVTCLACHTSFCVKCGADYHAPTSCDTIRQWMTKCADDSETANYI
SAHTKDCPQCHSCIEKAGGCNHIQCTRCRHHFCWMCFGDWKSHGSEYYECSRYKENPSVAAEANHVKARR
ALEKYLHYFERFENHSKSLKMEEELRDKIRKKIDDKNSNSPKLRDLKLEKLSIFLGSDFSHVTSFQVNEH
NGTWIDWQYLHKSVSLLTKCRYTLQYTYPFAYYLTASPRKNLNRTILPRIWLPEFSNFENRNSKLQKSSI
PSLIYDFEYQQAQLEKEVEELAWAVERADGTARGALEAHMHRAEHKRQTLLHDFFF
Caenorhabditis sinica   Csp5_scaffold_00429.g11261.t1
MSFQYSDYDDEDSDMEPVDEEEETMKFLTHDELESEMKASVSDVESVLQITHGVCRILLHKFKWNKDALF
DRFYDNPDTHAFLVSAQVIPKNKNPQSAVVTRKNGECDICCDVGELSGLACAHLACSECWKAYLTEKIVD
GRQCEIECMASKCTLFVEEEKIHSYINDPVVLAAYEQHKVNSYVMTNPYTKWCPGLDCGRAVKTTSTTAQ
LISCPCGARFCFCCGQQWHEPITCEYLKLWIQRCKDDSETFNWIVANTKDCPKCHVTIEKNGGCNHMSCR
SQTCKYEFCWLCMGDWKGHSDRGCNAYNESEKEKEKNREQSRASLNRYLFYYNRYMTHKQSLQLEKKLDC
QVALKQEQLQEEPVSMTYVEVKFLEKAVQILSECRETLMHSYAFAFYLKKDNNSLIFEDNQKDLELATET
LSGLLERDMEIDLEEEIDFRILVQTIQDKARYVEARRKVLLEHCVEGYEKGFWNLTK

Caenorhabditis sinica Csp5_scaffold_00429.g11260.t1
MYSDSEDIEMTGSGSESGDDTNQDYQILSRDELEEEMKIAIIDVQSVLQVDSESCKILLQKFKWNKDSLL
ERFYESPDTDAFLMENQVIPKETETVAEEGDADCDICCDFTELSGMVCNHRACKECWKMYLMDKIKEGQC
SIECMSASCKLLMAEDGILKYIDDQTAIDKYHKLVVDSYVDINSLIVWCPKEECGLAVKVQNSEPQLVTC
SCNTSFCFSCGLDPHDPAQCRHLKLWLKKADEMKERNHNGEAHGTDSDTFKWIMSNTRDCPKCLVSIEKN
GGCNYMQCRNNKCRYQFCWVCMNAWSVHSQAWYQCNSYNEDADKKREASREDLHRYLFYYTRYFNHKRSL
ELEQKLRRTVKKKMEKMEARQMRWIEVQFLEQAVNTLSQCRHTMMYTYAFAYYLKRDNNALIFEGNQKDL
ERDLDKEDVFTLKQWIQDKCRYVEHRRKILLDHCTEGYEQDFWEFSQ
Caenorhabditis sinica Csp5_scaffold_00497.g12116.t2
MSNEHEENILMEEIDFEQEVVEMEILSPVDLNIQMSTIILSVQSILPLSHGTIRVLLQKHKWNKDLLLEK
AFSEDQIFTESKITVIQDDEDYCNICCSEGAVLFSLGCAHVACSGCWGSYLTENIKNGKSEIQCIGDCER
LVGDEDVVRLLEFKDSSILSSYKNLTLKNYVDSSKSMVWCIGPDCQYIIQSENPGPHTVTCSCGTQFCFQ
CSQGPHDPVSCQNLKMFLKKSLESRPAGGTYTSDHNTNSWILSNTKDCPKCFTAIQKNGGCNMMTCKNPR
CRFVFCWLCLKMYSGHGLKPCNGFNIAEEKNRINSRAELLRFNFYFNRFKAHKQSLELEKKLVSTVNTKL
EKLQANGMSWTDTRCLSDAVRVLLKCRQTLLFTYPFAYYLERTDHALMFESNQKDLEMATEQLSGFLEQD
MEVESLLTLTQKVQDKCRYVEHRRKILLDHCAEGVTVDTVGLVEIKEIKVDVKPYMRVVFEFVLLFVLLV
ALVLVIVFFLFELVNWLFFKKSV
Caenorhabditis sinica Csp5_scaffold_00497.g12116.t1
MSNEHEENILMEEIDFEQEVVEMEVKTDKNEITILSPVDLNIQMSTIILSVQSILPLSHGTIRVLLQKHK
WNKDLLLEKAFSEDQIFTESKITVIQDDEDYCNICCSEGAVLFSLGCAHVACSGCWGSYLTENIKNGKSE
IQCIGDCERLVGDEDVVRLLEFKDSSILSSYKNLTLKNYVDSSKSMVWCIGPDCQYIIQSENPGPHTVTC
SCGTQFCFQCSQGPHDPVSCQNLKMFLKKSLESRPAGGTYTSDHNTNSWILSNTKDCPKCFTAIQKNGGC
NMMTCKNPRCRFVFCWLCLKMYSGHGLKPCNGFNIAEEKNRINSRAELLRFNFYFNRFKAHKQSLELEKK
LVSTVNTKLEKLQANGMSWTDTRCLSDAVRVLLKCRQTLLFTYPFAYYLERTDHALMFESNQKDLEMATE
QLSGFLEQDMEVESLLTLTQKVQDKCRYVEHRRKILLDHCAEGVTVDTVGLVEIKEIKVDVKPYMRVVFE
FVLLFVLLVALVLVIVFFLFELVNWLFFKKSV
Caenorhabditis sinica Csp5_scaffold_00732.g14925.t1
MSFLPTTTIRPTTERWFGFDVGDYYYYDGGPKVSWLNGGSKSEELLRFENFQAQLAMIIDLGASVLGIIF
NIFHLFILLNKELRSTVVFILMIGAGFSDIIVFCASILDKNYEYKARRGFVKGFYGSGEQWWLLFFVLVS
QGLQKFGRLLSSTLGLFMAIIRTVSVMFPMSTVADKLMKAKTGIILITVFAIITGGLYLDYYSRLEITLQ
KGVFYASDQISNLGPHIYIEAYLVFGLSLLYLAITVALMVALVAAQKRRKRLQGNDEKKGGDHTTLLVFL
MASMFFISLLLYSTFFVLGYYNRQHPDAVVIQVTDTLCLIAKTVLTLNSIFHCFICYFLSSQYRKVVDRF
LKLDRLNCGFKKKLKVKIVEPATRTYDDKLVETVKESGGSNRTY
Caenorhabditis sinica Csp5_scaffold_00775.g15366.t1
MKSLLVAIFFLNFCASEFPDELAKLVKLHACDPKCTFNHSEITLKTVEFFPECEEICGILKINENLDLID
DELGEVFRKMHTLHGGLIVENTQLTNLNFFSTRNLYGAIHFFCETFGILLKNNQNLKDISVFNKFYLWGD
DDYNDCSFKIENNPALSGKFLEEDYYKWHYWSFEGSQNLADFGCRGDQITQNTLKNYKNCTTFYGGLQLV
NITDTSEIDALSGIKKLYGGLEIQNTTLRDLSFLGKLNFLSVQNIGRRKKIAFNIHNNPNLKRLGAILTN
GITFEWYYNRVGNLENLHPDFCITVEEMVEFLRVNIYFANLHARYCKDTGNMQGAYFCRFEKMKKLDDDC
TYILGNILVDSGDEKFVGKFQYVTHIFGSITVINTTLESLDFLPNLNHIASLDESKPPIQIIGNLNLKKA
DLTSIQRQQNIFRLRNNFHIFVLISQCTSXXYIRSATPQPITSTTPKWHGREYGDYYYYDGGPWKDWDWK
MRYDYIIEENTRKQTMDTTETIDYIFSFLGLFLNLIHLFVLSRKDLRSGVVFIIMIGFIIMIGICLSDIL
VSSSSITESYCGNSADIFFEEGRCGKDLQYWFTVIELVSQAVQKFGKFTSASLTLFMTLIRTISVLFPMG
SLSAFLMKPKTGIFIGVGTGLFFMTWCIQYYRYYRFVLNSRICYEFISYDLTNMSQMQYVLEGCTLVALS
VIYMLVTVTLLIALRIVKRRKKSLGTAKSDNTSTLVVTMAILFFISVLFFSTVHILGNWSFGNDINKLSS
MLKYAANSVLKLNSILHCLICFLMSSQYREVVKKLIGFDKWNMVILEAVESSKKLHEVLKNVGKRYNVWE
LSDAAKRDILLRIKHRDWAALPETEVEKIKTENAKNLVHHFLEVSSSENKYFEGSLKWISSIMSPNMTAQ
LCGGQPFNAYQYTKYMSDSGAHYKKTADKTYPLYEVVKTPRDTLQVNMKFTQMDSFSHNLYMHYDVTLNL
TFIRDRNESVFSITHINQGGTCLDNGTYRYSTLKEDSEFLDHLKSHPDALILMNLFTPKIFLSSQSATPK
KIPDFWLSGLDSRGAFIEVCHDGETDVVRYSNDQFRSWYKRFRMMWHPVSEDYVKIQTENANEDTVVARI

TMTLQMGNLNGTHDWDFKISIKRNLHGDQKWYINQFEVKCAPTVDYKDQSLRVIRDIVIDEFLESVENLP
RPIPWYSSVEFVKEFTKNGTVDVEFCDAQIRTNLTQIQFHLYHQTKLRDGFIFTVILDSKSIMLTAEKSS
YFNMKTLSSPDNRPPYDYVYEHEWHFELKWDFWDQFYYIKRLQIGCGSEISKKAPNFLRLSLCGLIEEKE
CIKALNSIYSENE
Caenorhabditis sinica   Csp5_scaffold_00819.g15764.t3
MYPIMEEIVMYLFLTTTTAGPTTVRWYGKEPGDFYYYNGGPDVFWKDEFEFDDQTTNKILEKSEIVDHVI
SFVGLFLNILHLIILTQKELRSAVVFILIIGICVCDILVFSASISERYFGKSDEKAMYGGFCGTEKQYWT
IFMEIISKEFQKFGRLSSSLFALCAAAIRAISVVLPMSSVTEKLQKSRSGIFIFLGLVVICGVRSGIHFS
KYTIFKGGNHPTSCYIYSDGSQDDYDFYRLQEGFTILSLIFFYVLVTGTLLIALAIAKKRRKNLNMKSES
STSLLVIMMSISFLLSELLYGLIFLFSDNLATNKDRAISQLAYMSAYISKTILNFRFDVSLFHLLLLIIP
ISRSCEKIVASGSESKDNNFDIDW
Caenorhabditis sinica   Csp5_scaffold_01025.g17688.t1
MSTEEVENEPPPAPIFDANRNQLVTSSEVGKKRNEELQLHGDTDESEESDNEEEEDDGGFEGIEDVETED
VGKEVGCEDVEEEIDDEEEDDKAAGDVATYDSDSENQKGNEVLNIYELESDVTAITTDVKNILDVSAGIS
QNLLQKYRWNKERLLEKFYGTTDINQFLVDHDLIPAPTAEFPEETTECVICFEESVLTGLACQHQFCFGC
WNSYLTEKIIDGGQSEIKCMQQECQLLFQEEQISFYIADPIVMSMYRRAIINNYVETNRWLKWCHGVDCD
KAVKVALTSTRHVSCSCGSSFCFSCNKDSHEPVNCRLLVHWMKNDDNESFKWILTNTKECPKCKAPIEKN
GGCNHMTCTNKICRYEFCWLCMGNWVGHDRNQCNIFPGGNDANRERTLANLQRFEFFKTRYMAHQQSLKL
ERELRASIIDKMDKLREFFALSTPEVIYLEKALNVLTQCRHTLMYSYVFAYYLEPNYNSVIFEGNQQDLQ
SAVEQLSEILEMKLEDDNLETLKQRVLEKYRYVEQRRKCLLDHCAEGEENDYWAYNE
Caenorhabditis sinica   Csp5_scaffold_01074.g18175.t1
MYTDDEDDILMTEESDFEQEEEEQEVKTDKNGNQILSPTDLFTQMSTIIQSVQSILPLSHGTIRVLLQKH
KWNKDLLLEKAFSEDQNFSESKITVIQDDEDYCDICCSAGAELISLGCAHVACRECWGSYLTENVKNGKS
EIQCIGNCKLLMGDEDVEKLLESENSSILSSYKNLTLKNYVDSSKSMVWCIGPDCQNIIKSENPGPHTVT
CSCGTQFCFQCSQGPHDPVSCQNLKMFTKKSQESRTLGDSYSSDSNTNSWILSHTKDCPKCFTAIEKNGG
CNKMTCRSPKCRFQFCWLCLKDWKIHGYMPCNSYNATDEKNRLTSRAELLRYRFYFNRFKAHEQSLELEK
KLIKTVTTKMEKLQKAGISWTEVRYLSEAVKVLSKCRRILMFTYPFAYYLERTNHAIMFESNQKDLEMAT
EQLSGFLEQDMETESMKTLTQKVQDKCRYVEHRRNIMLDHCAEGVTVDTVGLVKMEEEITVKSSNEMLLR
EGAELLLTFVVLFALSMFFIYILRKLFGS
Caenorhabditis sinica   Csp5_scaffold_01694.g22580.t1
MFKISTTTTPNLITTMRWYGRDPIDYYYYEGGPDPGWASQPENFFTPRIQEKILENSEVVDHIFSYFGLI
LNILHFFILIRKELRTNVVFLIMIGICLCDLMVFSASIAEKKLHSEVGDSETCNTKFHWWMLFAEVFSKG
AQKLGRLSAAFLALSMAGIRAITVMFPMSTVSEKIMKEKFGAVMVLVEFLGCGIWYAVFYSNFSIEFFGS
NAFGMGGCYWVKDWSGGLYEYLEGFIVCILTCLYVIATATLIITLKHVQKKRKNLRGQDDKHSNTSLLVT
IMAISFFIAELVYSLIFLFSSQAILRSSNAVRLPIYTAALISYYLSVLNSASHCVICYFWSIQYRDTENV
AKPASCSERAATILTLPDSEH
Caenorhabditis sinica   Csp5_scaffold_01694.g22580.t2
MFKISTTTTPNLITTMRWYGRDPIDYYYYEGGPDPGWASQPENFFTPRIQEKILENSEVVDHIFSYFGLI
LNILHFFILIRKELRTNVVFLIMIGICLCDLMVFSASIAEKKLHSEVGDSETCNTKFHWWMLFAEVFSKG
AQKLGRLSAAFLALSMAGIRAITVMFPMSTVSEKIMKEKFGAVMVLVEFLGCGIWYAVFYSNFSIEFFGS
NAFGMGGCYWVKDWSGGLYEYLEGFIVCILTCLYVIATATLIITLKHVQKKRKNLRGQDDKHSNTSLLVT
IMAISFFIAELVYSLIFLFSSQAILRSSNAVSCNPSPNSTEVIVLPYQIFDIIGIRERLGPVLRVIPIFI
YPFLWIFLFIKFRKNQTQGSSEVRNSAKLLLIILTLTIFSDGASAIFGFVWIFVRRGHTNIELKLPIYTA
ALISYYLSVLNSASHCVICYFWSIQYRDTENVAKPASCSERAATILTLPDSEH
Caenorhabditis sinica   Csp5_scaffold_01694.g22579.t1
MFRISTTTTPNPLTTKRWYGRDPLDYYYYYEGGPNIGWVTLPDPWFNQKTEHDIIRISWVLDRFASYFGI
FLNILHFFILTRKELRTSVVFLIMIGICLCDILVFLASIIETETYTRYAEEFELSLMESYWMVLMNIITK
GLQKYGRMYSAVLALSMTVIRTITVMFPISVLSEKIIKTEFGVILILVEFLACGIWCAFFFSEYEIRKNN
TEISESQNFLLFSDFFFATSLFNRDETFFEKVERFTYCLLAAFYVITTSTLIITLKTVQKKRRRLGQDDK
GSNTTLLVTAMAISFLIAALVYSLVYFYGSRSHVPSIELFQLSRMFKTTSKTLLTFNSIFHCLLSFLLSS

QYRDVVKRIIRWNKISKGKRSGPNSSLAQVVTY
Caenorhabditis sinica   Csp5_scaffold_02000.g24308.t1
MNSDRALGSPPSKKSREMDSDISDYEQTDDYEQEEMDESFNMASDDDDAATEQADRDKLVSENQFLTQTD
LEFEMENLTQEVHGILEVSPGICRILLQKYQWNNNRLMDRFYESGDTKKFLIESCCLPKTFGATSSEGDC
DICCDTGELISLGCGHMACAECWKAYLTEKIKDGHWEIECLAPNCKLVMEEDKIMSYLKDPAIVATYHRV
IVNNYVATNSLLKWCPGADCGKAVKVTHMDPQLVVCPCGTRFCFSCCEDFHEPISCRLLKLWFKRCMDDS
ETSNWLSANTKDCPKCNVAIEKNGGCNHMRCTNKNCQFYFCWNCMKSLTNHQSCNRFESGDEQLTKNQAR
ARLEKYLFYYNRYMGHQKSLELEEKLKAIVAFKMDELQNFLMSWVEVQFLKEAVEVLRDCRRTLMYTYAF
AFYLKKDNNSEMFETNQNDLEQATEEISGLLERDLEDNDLYTLKHNVQNKCRYVAHRRKILLDHCTEGYE
QDTWKFTE
Caenorhabditis sinica   Csp5_scaffold_02026.g24451.t1
MSFDAEMDYDDGSSSEYQEEIEDKILDLPGIQSLMKSSISEVQPALEVSEGVGRILLQKFKWNKDPLMDR
FYETSDIQEFLKTLSVDSWKVETPSDSPEAPESKKLKTAEEDSQECDICCDVGDLSGPSCSHLSCPSCWH
SYISERINEGQSEIECMASKCKLIMPDELVLTYIKDDLKTLASFNRLIINNFVQSNTHLKWCPGVDCGRV
VYSSSTDPHVVLCDCGERFCFSCGNDGHEPVNCKHLKLWIKKSLDDSETSNWLNANTKDCPRCSVPIEKN
GGCNYIRCTNKTCLYQFCWICMNAWTVHAQAWYNCNAFVPTEATASAELARDQAKAILERYLFYFHRYRG
HQQSLEMEKKLIAKIEEKMEELQQDATMFWNDVLFLRQAVDVLSQCRRTMMYTYVFAFYLKKSNQSTIFE
NNQKDLEMATEQLSGFLERDLEDEENRDLKTLKLKVQDKCRYIQHRRKVLLDHCAEGVEQETWEYIE
Caenorhabditis sinica   Csp5_scaffold_02436.g26487.t1
MEQLQQLNMTWNEVQFLRQAVDTLSDCRRTMMYTYVFAYNLNRNNHSIIFESNQKDLEMATEQLSGFLEQ
DMEEEENLTSLKHKVQDKCRYVEHRRKVLSEHCAEGVEQGVWDFIEAK
Caenorhabditis sinica   Csp5_scaffold_02698.g27595.t1
MFPVSTTIIPPSSETTVNWYGPLPGDYYYFKGGPRENWYDEVVDIVPVDIQDQFNKKSEVVDHASYYFGL
LFNIPHLLLLCQKELRSHVVFIIMIGIAISDLLVFTASISQKYIGDTYTKAYMQGFCGTDRQWRLMLLEW
IAKGIQRMGRTNSALLALSMAGIRATTVTFPMSRVAGKLMEAKIGIVITVLLSMVSMVWYGTFYSRWSFD
SGKVHDLDCYNPSNEFEYLYTLIDGYTVLTITILHAITTVALLVALQVAKNRRRNLNNDKSANTSTLVTV
MAISFFISQLLYSLVFVFGQRGDTVDPSSITIIHFFAMFDNVSRTVLTLNSILHCFLSYFLSSQYRNVVK
RLMCSWRSKKNDSQNVNSVVVRTTNT
Caenorhabditis sinica   Csp5_scaffold_03210.g29432.t1
MSDYEDESDFDMMSEGEGIADIEEVTAVEDEFLTASQLETEMNGTISEVHSVLQVPLGMCRILLHKYKFN
KHALMDTFYESPDANAFLVAAKVIPESKATSSSGVSSSGGECEICCDDSGELSGLACGHMACGECWKGYL
TEKISSGGVGEIECMAAGCKLLVEDEKVLNYITEPNLIAAYRRRVVESYVETSKELKWCPGADCGRAVKI
THYEPHPITCQCGCTFCFTCGSNWHAPIECRHLKLWERRCMDDSETYNWIHANTKDCPKCHGAIEKNGGC
NRILCRGCNYQFCWLCMRDWDVHGYSKCNSFESKDAIDARQKSRYSLDRYLFYYNRFVQHQKSLKFEEKL
KVTIREKMEQLQNNSMSWIEVQFLPQAVETLNICRRTMMHTYAFAFYLQKDNNAMMFESNQSDLEMATEQ
LSGFLEQELVLEMDDMKSLKMKVQDKCRYVEHRRQLLLNHCAEGTEQGIWKYNEKN
Caenorhabditis sinica   Csp5_scaffold_03723.g31096.t1
MDSDGDMMDEQENIYSDMEHEDGSDIEMEDSTSDIEPEEKAKDEILDLASLESGMKEAITEIKETLCVSA
GVSRILLQKFKWNPEELKDKFYESSDIGEFLKKHQLSLEDSESYKVSEGVKESESQEDTECDICCDTGKL
IGLSCGHLACETCWKFYLAEQVKEGKSLIECMATKCDLLMPDEKVLELLESSQTYNNLIMNHYVQTNIFL
KWCPGVNCGKAVKSEHVDPHLILCTCGTRFCFSCCNDWHDPINCRQMKLWVKKCGESSEDATWIIKNTKD
CPKCLTSIEKNGGCNYIRCVNPKCGFQFCWICMNDWMVHKNAWYNCNSFDQNKETDRKEYRSNHDRYLFF
YNRYSTHEQSLKLEKK
Caenorhabditis sinica   Csp5_scaffold_07796.g38150.t1
ILTSTDLLNTMRSIIQNVQSILQVSPAVCRILLQKFNWNKDMLLERFYESPNFLVDYCIVPKDEASPKEY
PHDPEEPQECEICCDFTKLVGLDCGHLACRECWKSYLEEKMKEGKSEIECMASDCKLLAPDDTLKTFLDP
KSIANFENLTIKSYVEVSKNLTHCPKEPCQNILKSQTPGAHCVVCWCGHQFCFSCGDEPHDPATCRHLKL
WKKKAEAEKNRTASEGFSSNNETFQWILSNTKDCPKCLTAIEKNGGCNRIMCRSKTCRFEFCWLCMRSWD
VHGYSPCNTFNADEEKSRIDARAELLRFMFFYNRFKAHEQSLLLEKKVRKFD
Caenorhabditis tropicalis Csp11.Scaffold462.g1520.t2

MAPETIASKGNTTLALNQNQIGEEIRKMVREVQNVIYIREGKCIILLQKFDWNLESLLEDYENAGHLREY
FQSHGVCRQEILVIDNGECSICCDEVSVMGFECEHFACSQCWKQYLNINIDKPRIGCIDPECLYVVCADS
LRELGGDVEVQSQVIRNDFVDRSPNIVWCPSKDCQLAVKSDSFDTVECRCGLLFCFRCRLDPHAPATCIQ
VRNWEKRDDFGVTADEKSFGWIIRNTKECPKCMSPIEKQGGCDHMRCRKCYYEFCWHCGGWWLLHDGGCR
QIDVRESWLGNRSNKRSSQYFSNLYKVHLEKLESEKLLRDKYPQSQDVINVLIRSRKTLMYSIIYESNFY
GTGSREFKAKQWELETAVDALFTVMRRKHTKNKTKEYSGRQKMWRRGTTHEGFFGLLQKR
Caenorhabditis tropicalis Csp11.Scaffold462.g1501.t1
MSSNYSIGVDRTASDPAYSHIDIIENMRESIQKVQKLMDISEAKCQILLQNYKWNVDSLWETLQAIDDKS
IFLASNGISESDIIEVKDTECSICCEEGTVLGLSCGHTACRGCWKQYLAQNIDQLRIGCISPSCHLVLCL
DSIISFGGNVFVHRNALIKSYLESNPQMIKCPASICPLTVFANKIRTVQCPCRLRFCSQCLQQPHSPATC
LQIQNWGKKNRKGVSDQKSFEWIIKNTKECPKCSTMIQKNGGCDHMTCRKCKYSFCWSCSVSWREHYNRK
CYKEDVSKTMDRLNSRSNNHFMFLFNKNQNKLDSEKQLDPQVNRHNLHTLLESRRVLMYSFVYEYYGNYG
GEFKKKQKKLEESVNAFSDLLKKGVESNKKLVGNHDACVFMRWLKEEPSKWMNLYKTYRYTPYNEALGFD
YFEQQAKVFKGISPAEQEKELKRNHMYPVIEIMVPLQKFISCIRKVNSGVKIENADSFCLLYPEANLSSF
ISPSIGEPGYDCETGYRNVSGLCMLITTMPPTIIPTIPTTIPTTTTTTTTTTTTPTMTTTTTTTAPPQHL
TSDRPGTRSYTTTMIPRPLIQTKEDADLERRLKKYLAEETDGDEPDEEKDEEYYYDYDSCSSISIIIPLV
FVSFWFI
Caenorhabditis tropicalis Csp11.Scaffold462.g1501.t3
MSSNYSIGVDRTASDPAYSHIDIIENMRESIQKVQKLMDISEAKCQILLQNYKWNVDSLWETLQAIDDKS
IFLASNGISESDIIEVKDTECSICCEEGTVLGLSCGHTACRGCWKQYLAQNIDQLRIGCISPSCHLVLCL
DSIISFGGNVFVHRNALIKSYLESNPQMIKCPASICPLTVFANKIRTVQCPCRLRFCSQCLQQPHSPATC
LQIQNWGKKNRKGVSDQKSFEWIIKNTKECPKCSTMIQKNGGCDHMTCRKCKYSFCWSCSVSWREHYNRK
CYKEDVSKTMDRLNSRSNNHFMFLFNKNQNKLDSEKQLDPQVNRHNLHTLLESRRVLMYSFVYEYYGNYG
GEFKKKQKKLEESVNAFSDLLKKGVESNKKLVGNHDACVFMRWLKEEPSKWMNLYKTYRYTPYNEALGFD
YFEQQAKVFKGISPAEQEKELKRNHMYPVIEIMVPLQKFISCIRKVNSGVKIENADSFCLLYPEANLSSF
ISPSIGEPGYDCETGYRNVSGLCMLITTMPPTIIPTIPTTIPTTTTTTTTTTTTPTMTTTTTTTAPPQHL
TSE
Caenorhabditis tropicalis Csp11.Scaffold462.g1501.t2
MSSNYSIGVDRTASDPAYSHIDIIENMRESIQKVQKLMDISEAKCQILLQNYKWNVDSLWETLQAIDDKS
IFLASNGISESDIIEVKDTECSICCEEGTVLGLSCGHTACRGCWKQYLAQNIDQLRIGCISPSCHLVLCL
DSIISFGGNVFVHRNALIKSYLESNPQMIKCPASICPLTVFANKIRTVQCPCRLRFCSQCLQQPHSPATC
LQIQNWGKKNRKGVSDQKSFEWIIKNTKECPKCSTMIQKNGGCDHMTCRKCKYSFCWSCSVSWREHYNRK
CYKEDVSKTMDRLNSRSNNHFMFLFNKNQNKLDSEKQLDPQVNRHNLHTLLESRRVLMYSFVYEYYGNYG
GEFKKKQKKLEESVNAFSDLLKKGVESNKKLVGNHDACVFMRWLKEEPSKWMNLYKTYRYTPYNEALGFD
YFEQQAKVFKGISPAEQEKELKRNHMYPVIEIMVPLQKFISCIRKVNSGVKIENADSFCLLYPEANLSSF
ISPSIGEPGYDCETGYRNVSGLCMLITTMPPTIIPTIPTTIPTTTTTTTTTTTTPTMTTTTTTTAPPQHL
TSENKKLHDDYDPKTSHTNERRCGSGEKVEKVSGGRDRWR
Caenorhabditis tropicalis Csp11.Scaffold462.g1519.t1
MAPTTVASKEKSTVVLNQNKIREEIRKMGREVQKVINISEGKCIILLQKFYWNLKSLLKDHENAGHTPLY
LRSHGVCQQDTQVVDNGECSICCDEVSVMGFECEHFACSQCWKQYLDMNIDKPRIGCIDPECLYVVCSDS
LEELGGDVEVQAQVIRNDYVERSPNIVWCPSKNCQLAVKSDSFDTVECQCGLLFCFQCRLDSHVPATCTQ
VRNWETRENFGFTADEKSFAWILKRTKDCPKCMSPIEKQGGCSHMCCRKCYYSFCWHCGGWWFEHGMACR
QIDLGLKRLGNQSNKRSSLYFSNLYKKHSVKLEREKLLRYKLKGNTLNVLLQSRRTLMYSIIFEFYFYGN
GFKEFKTKQKELETSVDDFSTLMRLRRTENIKLEEVLDYELCFNALNTKFKDEFKEYRWMAFNYTTTRGI
DYFKNLTDTIFFNTFYDVGRSDDHGIHHAVMELEHVVPEQKQYCCANFTSPRFNLSNVFCVFGPEGTLDS
WFEKIEGTPGEECVHGYENNDGLCALIPATTSTISTTLPETTTTEPLTLPESTVTDLETSTSTTKFSETT
ELPIHLESRDHETTEMTKKLPGTTGLPIHLESQDHKTTEMSDLEKRVQKYRETETDGDEPDVEKEDEDEY
YDDSSSSIFSFSLIFVILLFFES
Caenorhabditis tropicalis Csp11.Scaffold470.g1631.t1
MDDEDMSCTSGEDYGGYGDEDYYNEADIDAADDVAVTPTQSEEADYECLSVNQVERVFIDGVNSLLSRIQ

INEKQARILLEVNQWDIDKIVSRYRKDRAEFLRKSHLETRPEQKRKHSTAVTKGYCSVCAMDGYTDLPRL
NCGHCFCEHCWKSHIESRVSEGVAARIECMESECEVYAPAEFVLIILRSSPSLKTKYERFLLRDLVNSHP
HLKFCVGNDCQVIIRSTEVKPKRVTCQCCHTSFCVKCGADYHAPTSCETIRQWMTKCADDSETANYISAH
TKDCPQCHSCIEKAGGCNHIQCTRCRHHFCWMCFGDWKSHGSEYYECSRYKENPSVAAEANHVKARRALE
KYLHYFERFENHSKSLKMEEELRDKIRKKIDDKVNEHNGTWIDWQYLHKSVSLLTKCRYTLQYTYPFAYY
LTAGPRKNLFEYQQAQLEKEVEELAWAVERADGTARGALEAHMHRAEHKRQTLLHDFFF
Caenorhabditis tropicalis Csp11.Scaffold564.g4005.t1
MDPPDDELFEDSDEFAEVDIPDNEEEEQADDDVPNDTDGFSDVDVSDNEEEEQAEDEEVNDEQEENSNDG
KMSGMSGIDPEPVAVDAEEVKFHTVAELTDEMDGLIEGCHNTLQLPRVVCRILLQMLKWDPVDVNSAPFD
LAKPPKIDQLLKDVKVPKVVTVDKTECCGDTNVLGLDCGHRACRECWERYLTEEIKEQKSDISCLAPDCK
CLVDDEKIREYLVLPEHLAVFDRLKLNKYVETNPKVTWCPGKCELALKVTDSDQKVTCPSCSKVFCAGCL
GDVHEPVPCNLLKEWQEEIEKSQALLPRDKKTCPQCKNMIGKTVGSHQCKCKCWHSFCWLCLNAWGDHDK
NPCAIYTPVKPRDNDRYLFHYIRFIEHNRSLVVEEKYKKTVVEVTKSIRKRISAAEIESLEKSVKLLSEC
RQIVKFGYPFMYYLKKRDRQFEVYQKNLATSVEQLTRFLKEKGGVLYNKAFFEALSDKCNDAETCRGILL
KYYRDGKKEEKFEYSNINN
Caenorhabditis tropicalis Csp11.Scaffold595.g5246.t1
MSSDDEIHMDDSDSDQEEIDDACLSDDDGIALESHEQTSKDYRDNAEPENEVLSHDQLEMEMKKTIADVQ
AVLQVKTGTCRILLHKYKWNKESLLERFYEHPDTTAFLIDAQVIPRHTETLPVGESECDICCMVSGLSGL
SCNHRACTPCWISYLTNKIVEGGQSEIECMAANCKLLIEDEKVMHYITDPTVIASYRRLIVASYVETNRL
LKWCPGVDCGKAVRVAHCEPRLVVCSCGSRFCFSCGNDWHEPVNCRLLKLWMKKCSDDSETSNWINANTK
ECPKCMITIEKDGGCNHMTCKNTACRFEFCWMCLGPWEPHGSSWYNCNRFDDSVAKTARDAQEVSRANLQ
RYLFYYNRYMGHQQSLRLEGKLYASVKTKMEQMQTQSMSWIEVQFLRKAVDILSECRRTLMFTYAFAFYL
KRDNNSIIFETNQKDLEMETEQLSGFLERDMDQENLVTLKQKVQDKYRYVEHRRKILLDHCAEGAEQDLW
VFNE
Caenorhabditis tropicalis Csp11.Scaffold608.g5815.t1
MSSDEDDLEYEEMETDKKEYEFLSFDQIRSEINDAITSVQSVIEAPNGVCRILLHKYKWNEDSLLERFYE
SPDTRAFLVAANVVPKETVSSSGANGQCEICFEEEEMVELACKHRACRSCWNGYLTQKIMDERMCEIKCM
MPDCQLIMEDEKIHEFISDPVVLATYEKLTVDSYVQASIFLKWCPGVECGRAVKLEDCDRHIVICPCGMM
FCSSCGNDTHEPISCGLLKLWLKRQEDDSETANWINVHTKECPKCHVAIEKNQGCNHMTCRECKHQFCWL
CMKPWSGHANCNRYEDAERIAQQSITRSNLERFLFYTGRYNSHRESLEHEKKLKETVEFKIRQLAMSDYG
WVDAQYLKRAVEVLADSRKTLMNTFAFAFYLEKDNNSIIFESNQSDLQLATESLSRILGSDLDVDQISAL
KSTIQDSCRYVDSRRVVLLEHCEEGVNKGFWNYCKRLL
Caenorhabditis tropicalis Csp11.Scaffold608.g5816.t1
MDSEDEIAFEDSDQEMSTVSGYEILDQSALESELKDLITDVQDVLEVSPGVAQILLMKFKWNKGALMEKF
YESQDANAFLADAQIIPKPKEGKPALRQGECAICCDETELTGLACGHESCSMCWEMYLVEKIKSGVCQIQ
CMASDCNLIMGVEMILSYLTDNEVIVKYRHLVLRNYVEANSVLGWCPGANCEKVVQVNYNEPQLIECSCG
TRFCFSCSNDSHKPATCHLIRQWKKKCQEMQEKKHTGEGYGTDSETFQWIMSNTKDCPKCFVSIEKNGGC
NYMQCRNENCRFQFCWICMKDWNVHRNGWYSCNAYDANADAKRESSRAEFHRFLFYYTRYINHEKSLKLE
ENLRKTIRQKMEDMEKLSLRWIDVQYLQKAVDTLSECRRTMKNTYVFAYYLKRDNNSLIFETNQRDLEMA
TEQLSGYLERDIDDTDFDTLRQNVLDKSRYVEHRLQILQQHCEEGKEQDLWEFVE
Caenorhabditis tropicalis Csp11.Scaffold629.g16127.t1
MSSDDEDVYMEDDSDQKDDDKLEFLDSEDLEEDMKMAIESVKIVLQVSSGVCRLLLQKYKWSKESLLDRF
YENQDTVSFLIDAHLLPGRSVAGCDQSSTECQICCTEGELSGLACNHLACKDCWNAYITDKIKEKHSEIE
CMASDCKLLIEDERVINYLEDPSIYQKVLVNSYVATNKSLRWCPGSCGKAVKVRSLADASIIICSCGVCF
CFSCGHEGHDPINCRLLKLWMKKCQEDNQTFNWINVNTKDCPKCSSPIEKNGGCNYMRCHACKYEFCWLC
FGYWKDEGAHSCNKFNETDNSSKRETCRISLEKYLFYYNRYLAHHRSLNLEQKLKDIVTTKMDHMQDLSM
SWVEVQFLQKAFEVLSECRRTLMYTYAFAFFLKRDNNVMIFEANQADLERSTEQLSGLLERDLDHDDLIG
MKQAVQDKFRYVEQRQKVLLDHCAEGKDLDIWKFNDGN
Caenorhabditis tropicalis Csp11.Scaffold629.g9130.t1
MLLRNVLSKGLRIRNSSSYCLRPGVGLERDVEDVLEAAHQFAKKEMYPKMAEWDKKGELPMDVLQKAGEM

GFGAIYCSGDHGGSGLSRLHASVIFEQLSMGCVSTAAYISIHNMCAWMLDTYGPDKLKEDLLPEMALFKK
LGSYCLTEPDAGSDAASIRTTATKKGDYYVVNGSKAFISGAGTSDNYFVMMRQDGAAPGAKGIFCLMIED
GTEGFSYGKKEDKLGWNSQPTRILTFEDCKVPITNQIGKDGFGFNIAMAGLNGGRVNIASCSLGAAQRSL
DLAIEHLKCRKQFGKTLADFQYNQFKLAELATKLYTSRLIVRAAAEQLDNDDPEKVALCAMAKLHATDNC
FEVVNGALQMFGGYGFLKDYPIQQYLRDIRVHQILEGTNEMMRLLISRDLLTKDIFWSSNSKSVLTMDSD
DEIHMDSGSEHEDEDQFFTSTELEEQMTEAISDVQDVLQASPGTCRLLLLKYKWNRDTLLERFYEDTSAF
AAPSAPKPVEQGECDICCDVTELSGLSCEHRACRECWKAYLTEKIVGGVSEIQCMATGCEQVMEDEKVMS
YVEDPKMVTMYKKLVLDSYVQANQFIKWCPGVNCGRAVKISNLDPHPILCPCGARFCFDCGTDCHEPVNC
RLLKAWLRRCEDDSETFNWISSNTKDCPKCFTPIEKNGGCNRIRCCKCSYQFCWQCMRDWDVHGYSACNS
YEEKSTVSGREKTRISLERYLFYYNRYHNHLKSLKLEMKLKAMVANKMLQMQDLGQGWVEVQYLHKAVEV
LRECRQTLTYTYVFAFYLQKDNNSQLFETNQNDLEMAVEQLSGFLEQDLENEDLVTLKQKVMDKCRYVEH
RRKRLIDQCREGTEQDIWVFKEEEIPEKKK
Caenorhabditis tropicalis Csp11.Scaffold629.g8801.t1
MYIFSTTTLKPLSTTKRWNGRDPTDYYFFHGGPDEYWDTRDDYLFEDGTVLKALDTTENIYYWASYIGLF
LNLLHFFVLTRKELRSNVVFIIMIGICFSDLLVFFSTISERYFGKSDEIGYREGWCGSDKQWWWILIELC
SQAIQKYGRLSSAILVFFMASIRSFSVIFPMSSMINILLKTRTGVITVLATWLFCGGWYWKYYAEYFIRK
PKKFKEPDNTSMLVVMMAVSFFISEVIYCFFFVMTDRDNDHDATILQLADLSEFISKTILILNSILHCFI
CFFLSSQYREVARRVLFLDRAPKRETAHPKTRSAAVGTTEMAKSSV
Caenorhabditis tropicalis Csp11.Scaffold629.g13772.t1
MMDLDEEYYMYKTDKSDDIQYEFLSSEDLKTEMTTIIENVKAMLQYPSGICRILLQQHKWSNQLLLDKFY
ENPDPNDFLAKCGLLPEGSVIVSEEQECPICCISGKLFGLACNHMACVDCWSAYIDGKMKAKRSEIECMQ
LDCKLLMVDEKVTEFIKDASIYEDVIINSYVKTHKTLRWCPETCRTAVRVKTPVHSLISCPCGVNYCFTC
GNEGHEPINCRLLRKWLRRCQDDNSSYKWLNANTKDCPTCQTPIEKTGGCNHMTCQKCKKHFCWLCLTPW
NLRHDCSR
Caenorhabditis tropicalis Csp11.Scaffold629.g8309.t1
MYLFLTTPFPRPSTERWFGFEVGDYYYYDGGPEAYWDQKPDYIFDDKTKDEIIEQSDIADLVVSFIGLFL
NFLHLLILTRKELRANVVFIIILGISICDILICIGSITERYFKDSHFIARYEGFCGTDYQWWMVFIEAFS
QGIQKFGRLTEALFALSTAAIRAVTVLFPMSSMADRLMKLTSGIIILVFNSVLCGVLYSIYYSMIEIYRE
GNSDL
Caenorhabditis tropicalis Csp11.Scaffold629.g8781.t1
MSSDDEIYMDSESEEEQEEEVEDQILDEKGLRADMKQQIAEIQSTVEVSEGVGRLLLQKHKWNKDSLTDK
FYESPDRITFLIESNVHPKECVPVETGEGDCDICCETTELVGLSCNHRCCKECWNSYLTEKIKEGQSEIE
CMDSRCKLLLDDEKVEEFLTDSSIIATFHRLILNKYVSSNVFLKWCPGVDCGKAVKSTHCDPHLVTCTCG
TGFCFYCTNEWHDPVNCHHMRLWMKKCGENAETATWIINNTKDCPKCLAQIEKNGGCNYIRCTNPACGYQ
FCWICMNSWSVHAQAWYNCSSFDQAAESNREKYRTNLDRYIFYYNRYRGHQQSLKLESKLIRKVEKKMDK
MQEKGMSFSDVQCLRHAVDVLSVCRQTMMLTYVFAYYLDKNNHSLIFEANQKDLEMATEQLSGFLERELE
DEDLTTLKQSVQDKSRYVEHRRKRLLDHCADGNEQNHWVFIE
Drosophila melanogaster NP_477374.1
MDSDIEMDMESDNDGEYDDDYDYYNTGEDCDVERLDPKRADPEYFEYECLTVEDIEKLLNERVEKLNTIL
QITPSLAKVLLLEHQWNNVAVVEKYRQDANALLVTARIKPPSVAVTDTASTSAAAASAQLLRLGSSGYKT
TASATPQYRSQMCPVCASSQLGDKFYSLACGHSFCKDCWTIYFETQIFQGISTQIGCMAQMCNVRVPEDL
VLTLVTRPVMRDKYQQFAFKDYVKSHPELRFCPGPNCQIIVQSSEISAKRAICKACHTGFCFRCGMDYHA
PTDCQVIKKWLTKCADDSETANYISAHTKDCPKCHICIEKNGGCNHMQCFNCKHDFCWMCLGDWKTHGSE
YYECSRYKDNPNIANESVHVQAREALKKYLHYYERWENHSKSLKLEQQTIDRLRQRINSKVMNGSGTWID
WQYLFNAAALLAKCRYTLQYTYPYAYYMEAGSRKNLFEYQQAQLEAEIENLSWKIERAETTDLGDLENQM
DIAEKRRTTLLKDFFPVDA
Drosophila melanogaster NP_523399.1
MDSDNDNDFCDNVDSGNVSSGDDGDDDFGMEVDLPSSADRQMDQDDYQYKVLTTDEIVQHQREIIDEANL
LLKLPTPTTRILLNHFKWDKEKLLEKYFDDNTDEFFKCAHVINPFNATEAIKQKTSRSQCEECEICFSQL
PPDSMAGLECGHRFCMPCWHEYLSTKIVAEGLGQTISCAAHGCDILVDDVTVANLVTDARVRVKYQQLIT

NSFVECNQLLRWCPSVDCTYAVKVPYAEPRRVHCKCGHVFCFACGENWHDPVKCRWLKKWIKKCDDDSET
SNWIAANTKECPRCSVTIEKDGGCNHMVCKNQNCKNEFCWVCLGSWEPHGSSWYNCNRYDEDEAKTARDA
QEKLRSSLARYLHYYNRYMNHMQSMKFENKLYASVKQKMEEMQQHNMSWIEVQFLKKAVDILCQCRQTLM
YTYVFAYYLKKNNQSMIFEDNQKDLESATEMLSEYLERDITSENLADIKQKVQDKYRYCEKRCSVLLKHV
HEGYDKEWWEYTE
Drosophila melanogaster   NP_648392.1
MNSEMEFSDEDHGDSHRSLLTHMSCENDSDSEDTCTEILLPENSNSPETEDFVYKVLSVDQIVQHQRNII
DEVNNVLNLPPQVTRIILNHFKWDKESLFENYFESNPKDFFQRAHVLNPFEKKIERESAASTSCAIPQLC
GICFCSCDELIGLGCGHNFCAACWKQYLANKTCSEGLANTIKCPAANCEILVDYISFLKLADDSEVVERY
QQLITNTFVECNMLMRWCPAPNCSHAVKAVCAEPRAVLCKCGHEFCFACGENWHEPASCSSLKKWVKKCL
EDSETSNWIAQNTKECPKCNVTIEKDGGCNHMVCKNPSCRYDFCWVCLGSWEPHGSSWYSCNRFDEEEAK
QARLAQQKYRSSMARYLHYYNRYSNHMQSLKMENKLYSNIQAKMDDMQEEMSWIEVQFLRDAVDVLCQCR
TTLMYSYVFAFYLMNNNQKIIFEDNQKDMEMATEKLSECLEREITVKNIYEVKQKVLDLSHYCQKRRHVL
LCHVREGYENDWWEFKEETTT
Homo sapiens   NP_006312.1
MSVDMNSQGSDSNEEDYDPNCEEEEEEEEDDPGDIEDYYVGVASDVEQQGADAFDPEEYQFTCLTYKESE
GALNEHMTSLASVLKVSHSVAKLILVNFHWQVSEILDRYKSNSAQLLVEARVQPNPSKHVPTSHPPHHCA
VCMQFVRKENLLSLACQHQFCRSCWEQHCSVLVKDGVGVGVSCMAQDCPLRTPEDFVFPLLPNEELREKY
RRYLFRDYVESHYQLQLCPGADCPMVIRVQEPRARRVQCNRCNEVFCFKCRQMYHAPTDCATIRKWLTKC
ADDSETANYISAHTKDCPKCNICIEKNGGCNHMQCSKCKHDFCWMCLGDWKTHGSEYYECSRYKENPDIV
NQSQQAQAREALKKYLFYFERWENHNKSLQLEAQTYQRIHEKIQERVMNNLGTWIDWQYLQNAAKLLAKC
RYTLQYTYPYAYYMESGPRKKLFEYQQAQLEAEIENLSWKVERADSYDRGDLENQMHIAEQRRRTLLKDF
HDT
Homo sapiens   NP_005735.2
MDSDEGYNYEFDEDEECSEEDSGAEEEEEDEDDDEPDDDTLDLGEVELVEPGLVGGERDGLLCGETGGGG
GSALGPGGGGGGGGGGGGGGPGHEQEEDYRYEVLTAEQILQHMVECIREVNEVIQNPATITRILLSHFNW
DKEKLMERYFDGNLEKLFAECHVINPSKKSRTRQMNTRSSAQDMPCQICYLNYPNSYFTGLECGHKFCMQ
CWSEYLTTKIMEEGMGQTISCPAHGCDILVDDNTVMRLITDSKVKLKYQHLITNSFVECNRLLKWCPAPD
CHHVVKVQYPDAKPVRCKCGRQFCFNCGENWHDPVKCKWLKKWIKKCDDDSETSNWIAANTKECPKCHVT
IEKDGGCNHMVCRNQNCKAEFCWVCLGPWEPHGSAWYNCNRYNEDDAKAARDAQERSRAALQRYLFYCNR
YMNHMQSLRFEHKLYAQVKQKMEEMQQHNMSWIEVQFLKKAVDVLCQCRATLMYTYVFAFYLKKNNQSII
FENNQADLENATEVLSGYLERDISQDSLQDIKQKVQDKYRYCESRRRVLLQHVHEGYEKDLWEYIED
Mus musculus   NP_035920.1
MSVDMNSQGSDSNEEDYDPNCEEEEEEEEDPGDIEDYYVGVASDVEQQGADAFDPEEYQFTCLTYKESEG
ALHEHMTSLASVLKVSHSVAKLILVNFHWQVSEILDRYRSNSAQLLVEARVQPNPSKHVPTAHPPHHCAV
CMQFVRKENLLSLACQHQFCRSCWEQHCSVLVKDGVGVGISCMAQDCPLRTPEDFVFPLLPNEELRDKYR
RYLFRDYVESHFQLQLCPGADCPMVIRVQEPRARRVQCNRCSEVFCFKCRQMYHAPTDCATIRKWLTKCA
DDSETANYISAHTKDCPKCNICIEKNGGCNHMQCSKCKHDFCWMCLGDWKTHGSEYYECSRYKENPDIVN
QSQQAQAREALKKYLFYFERWENHNKSLQLEAQTYERIHEKIQERVMNNLGTWIDWQYLQNAAKLLAKCR
YTLQYTYPYAYYMESGPRKKLFEYQQAQLEAEIENLSWKVERADSYDRGDLENQMHIAEQRRRTLLKDFH
DT
Mus musculus   NP_064311.2
MDSDEGYNYEFDEDEECSEEDSGAEEEEEDDDEDEPDDDNLDLGEVELVEPGLVGGERDGLLCGETGGGG
GSALGPGGGGGGGGGGGGPGHEQEEDYRYEVLTAEQILQHMVECIREVNEVIQNPATITRILLSHFNWDK
EKLMERYFDGNLEKLFAECHVINPSKKSRTRQMNTRSSAQDMPCQICYLNYPNSYFTGLECGHKFCMQCW
SEYLTTKIMEEGMGQTISCPAHGCDILVDDNTVMRLITDSKVKLKYQHLITNSFVECNRLLKWCPAPDCH
HVVKVQYPDAKPVRCKCGRQFCFNCGENWHDPVKCKWLKKWIKKCDDDSETSNWIAANTKECPKCHVTIE
KDGGCNHMVCRNQNCKAEFCWVCLGPWEPHGSAWYNCNRYNEDDAKAARDAQERSRAALQRYLFYCNRYM
NHMQSLRFEHKLYAQVKQKMEEMQQHNMSWIEVQFLKKAVDVLCQCRATLMYTYVFAFYLKKNNQSIIFE
NNQADLENATEVLSGYLERDISQDSLQDIKQKVQDKYRYCESRRRVLLQHVHEGYEKDLWEYIED

Pristionchus pacificus   PPA01369
MVVLTGLRCRHYACKPCWERYLTTKVLVDSTSIIECLYPDCKLLVDDEHFMTLTGKNNVVKKAFSRLTLN
NFVESNRKLRWCPGTGCEMAVKVTSDQTRAVECTCKCRFCFECGKEWHEPMDCALLRKWLQKCVDDSETS
NWISANTKDCPKCHTTIEKNGGCNHMTCKSPACTYEFCWICMGDWKAHKDAYNCSRYVESGTVKDSRAAL
EKYLHYYNRYMNHQNSLKLENKLYDSVRAKMDLMQRVSTMSWVEVQFLLKAVDVLSECRRTLMFAYAFAY
YVKQGNELFIFEDNQRDLEIATEQLSGFLEQDLDQILQTENMVTLKQKVQDKYRYVEHRRNLLLRLLSCL
LAAFGHAAAGSNSAASVAGAAGSSSSAGAAVPAARAASSPGLGAPPGGTPGVGDGAPGAAAGGVVANNPA
TTTAHPPIVLPPAAGLTIDYETCVRCARELSDEV

Pristionchus pacificus   PPA29960
MIIRSREQKPRRIVCDKCTSTFCATCTGAYHAPTSCEMIKKWLVKCADDSETANYISAHTKDCPQCHSCI
EKNGGCNHMQCLKCKHHFCWMCFGDWKTHGSEYYECSRYRENPEVATEANHMKARRALEKYLHYYERYEN
HAKSLRMEMQMRDKLKQKIEDKVTCHDGTWIDWQHLHEAASLLAKCRYTLQYTYPFAYYLEKCARKELFH
ASVAEKASVVLTSLSSH

Schmidtea mediterranea   mk4.008683.00
MEFKSHYLLDDMTGLGCGHIFCLGCWVRYLNQKIVNENQGDQILCPAHQCDIIIDDSMVYNLIRDHPDIK
QRFQITVANSFVNCNPLLTWCPGVECNHIVRVARREPIRIDCSSCSSVFCFACGESWHDPVQCSFLEKWL
KKMRDDSETSNWIMANTKECPKCRATIEKNGGCNHMVCKNVACKYEFCWVCLDQWEPHGSAWYNCNRYNE
TDAQKARDAQEASRAALQRYLFYFNRYANHLQSLKFESKLYTGVQKKMEEMQQHNMSWIEVQFLKQAVDV
LCDCRRTLNYTYVFAYYLKKNNQSIIFEGNQSDLEQSTEQLSEYLERDITTDSLVDIKQRVQDKTRYCKQ
RRAVLLKHVHEGYENDWWEHLDL
